# Preliminary Anatomical, Histological, and Histochemical Study of the Orbital Gland Complex in Kirk’s Dik-Dik (*Madoqua kirkii*) and Red Forest Duiker (*Cephalophorus natalensis*)

**DOI:** 10.3390/vetsci13080752

**Published:** 2026-07-29

**Authors:** Joanna Klećkowska-Nawrot, Aleksander Chrószcz, Abit Aktaş, Karolina Barszcz, Wojciech Paszta, Karolina Goździewska-Harłajczuk, Dominik Poradowski

**Affiliations:** 1 Department of Biostructure and Animal Physiology, Division of Animal Anatomy, Faculty of Veterinary Medicine, Wrocław University of Environmental and Life Sciences, Kożuchowska 1, 51-631 Wrocław, Polandkarolina.gozdziewska-harlajczuk@upwr.edu.pl (K.G.-H.); 2 Department of Histology and Embryology, Faculty of Veterinary Medicine, Istanbul University-Cerrahpasa, 34320 Istanbul, Turkey; 3 Department of Morphological Sciences, Faculty of Veterinary Medicine, Warsaw University of Life Sciences, 159 Nowoursynowska, 02-776 Warsaw, Poland; 4 Wrocław Zoological Garden, Z. Wróblewskiego 1-5, 51-618 Wrocław, Poland

**Keywords:** Harderian gland, superficial gland of the third eyelid, lacrimal gland, Kirk’s dik-dik, red forest duiker, Bovidae, histology, histochemistry

## Abstract

The orbital glands are part of the ocular surface protective system, but their morphology and secretory profiles remain poorly documented in small wild bovids. This preliminary descriptive study examined nine young animals: four Kirk’s dik-diks (*Madoqua kirkii*) and five red forest duikers (*Cephalophorus natalensis*). All animals underwent gross anatomical examination, and bilateral Harderian glands (HGs), lacrimal glands (LGs), and superficial glands of the third eyelid (SGTE) were examined histologically, histometrically, and histochemically. No inferential statistical comparisons between species were performed. In both groups, the HG was located medioventrally, the LG dorsolaterally, and the SGTE medially near the third eyelid. The glands showed compound tubuloacinar or predominantly acinar organization. Descriptive variation involved capsule thickness, septal development, lobular arrangement, secretory epithelial appearance, and plasma cell and lymphocyte distribution. Histochemical reactions demonstrated mixed secretory profiles containing neutral glycoconjugates and acidic mucosubstances consistent with sulfated and/or carboxylated components. Their relative proportions varied among glands and between groups. These findings provide preliminary anatomical and microscopic reference data for rarely examined wild bovids. Because of the small sample sizes, the observations should not be interpreted as definitive species-level differences.

## Simple Summary

Orbital glands contribute to the stability of the precorneal tear film and protect the ocular surface, but their structure is poorly documented in many small wild bovids. This preliminary descriptive study examined nine young animals from the Wrocław Zoological Garden, Poland: four Kirk’s dik-diks (*Madoqua kirkii*) and five red forest duikers (*Cephalophorus natalensis*). In every animal, the bilateral Harderian glands, lacrimal glands, and superficial glands of the third eyelid were examined macroscopically, histologically, histometrically, and histochemically, and all staining procedures were applied to all specimens. The study did not include inferential statistical comparisons between species. The three glands occupied distinct orbital regions and showed variation in capsule and septal development, lobular organization, secretory units, and histochemical reactions. Neutral glycoconjugates and acidic mucosubstances were present in different proportions, and plasma cells and lymphocytes occurred in the glandular interstitium. Because the groups were small, these differences are reported as preliminary observations from the examined animals rather than as established species-level characteristics. The results provide reference material for comparative anatomy and the microscopic assessment of orbital tissues in zoo and wildlife medicine.

## 1. Introduction

The ocular surface is protected by a closely connected system of anatomical structures, including the eyelids, conjunctiva, third eyelid and orbital glands. Among the accessory organs of the eye, the Harderian gland (HG), lacrimal gland (LG) and superficial gland of the third eyelid (SGTE) play an important role in maintaining the precorneal tear film and protecting the cornea and conjunctiva [1,2,3,4]. Their secretory products contribute to lubrication, hydration, mechanical protection and local defense of the ocular surface. For this reason, the orbital glands should be considered not only as anatomical structures, but also as components of a functional glandular system involved in ocular surface homeostasis. In mammals, the HG is usually a multilobar tubuloacinar gland located in the medial region of the orbit and associated with the third eyelid. Its size, topography and secretory character vary considerably among species [1,3,5,6,7,8]. In many mammals, the HG contributes to the precorneal tear film and supports lubrication and protection of the eyeball and third eyelid [1,3,9]. In some groups, especially rodents, additional functions connected with photoprotection, pheromone production, endocrine activity and reproductive regulation have also been described [3,5,8,10,11,12,13,14]. The SGTE, also known as the nictitans gland, is closely associated with the third eyelid and usually produces a mucoserous secretion. Together with conjunctival goblet cells, it contributes to the mucin component of the precorneal tear film and helps maintain a stable, hydrated and protected ocular surface [1,2,9,15,16]. The LG, located in the dorsolateral region of the orbit, is mainly responsible for the aqueous component of the precorneal tear film. It is usually described as a compound tubuloacinar gland producing serous or mucoserous secretion, depending on the species [1,4,7,17,18,19,20]. Its secretion nourishes and lubricates the cornea and conjunctiva, contains protective factors and supports the stability and repair of the ocular surface [2,15,18,21,22]. Histological and histochemical evaluation of these glands can therefore provide more than a structural description. The organization of secretory units, connective tissue stroma, ducts and immune-cell populations may help define the normal microscopic background of the ocular adnexa, whereas histochemical reactions allow the localization of neutral and acidic mucosubstances that contribute to the character of glandular secretion. Such information is useful when normal glandular architecture has to be distinguished from *post-mortem* change, age-related variation, or early pathological alteration in zoological and wildlife material. Although the orbital glands have been examined in several domestic and wild mammals, detailed data on their structure in wild ruminants remain limited. Previous studies have described selected ruminant species, including the one-humped camel, alpaca, roe deer, American bison and European bison [9,15,17,18,19,20,23,24]. However, many wild ungulates, especially smaller or less commonly examined species, are still poorly documented. This gap is particularly relevant for small bovids kept in zoological collections, where clinical ophthalmic interpretation often depends on limited comparative anatomical material. Such information may be useful not only for comparative anatomy but also for veterinary ophthalmology, especially in zoological garden animals and exotic ungulates.

Therefore, the aim of the present study was to provide a preliminary descriptive analysis of the orbital gland complex in two small wild bovid species from the Wrocław Zoological Garden: Kirk’s dik-dik (*Madoqua kirkii*) and red forest duiker (*Cephalophorus natalensis*). The study addressed the gross topography, microscopic organization, stromal framework, secretory units, histometric features, and histochemical secretory profile of the HG, LG, and SGTE. The observations were intended to define the features present in the available material and to provide reference data for future studies of ocular adnexal morphology and pathology. No inferential comparisons or functional conclusions were planned.

## 2. Materials and Methods

### 2.1. Animals and Study Material

**Figure 1:**
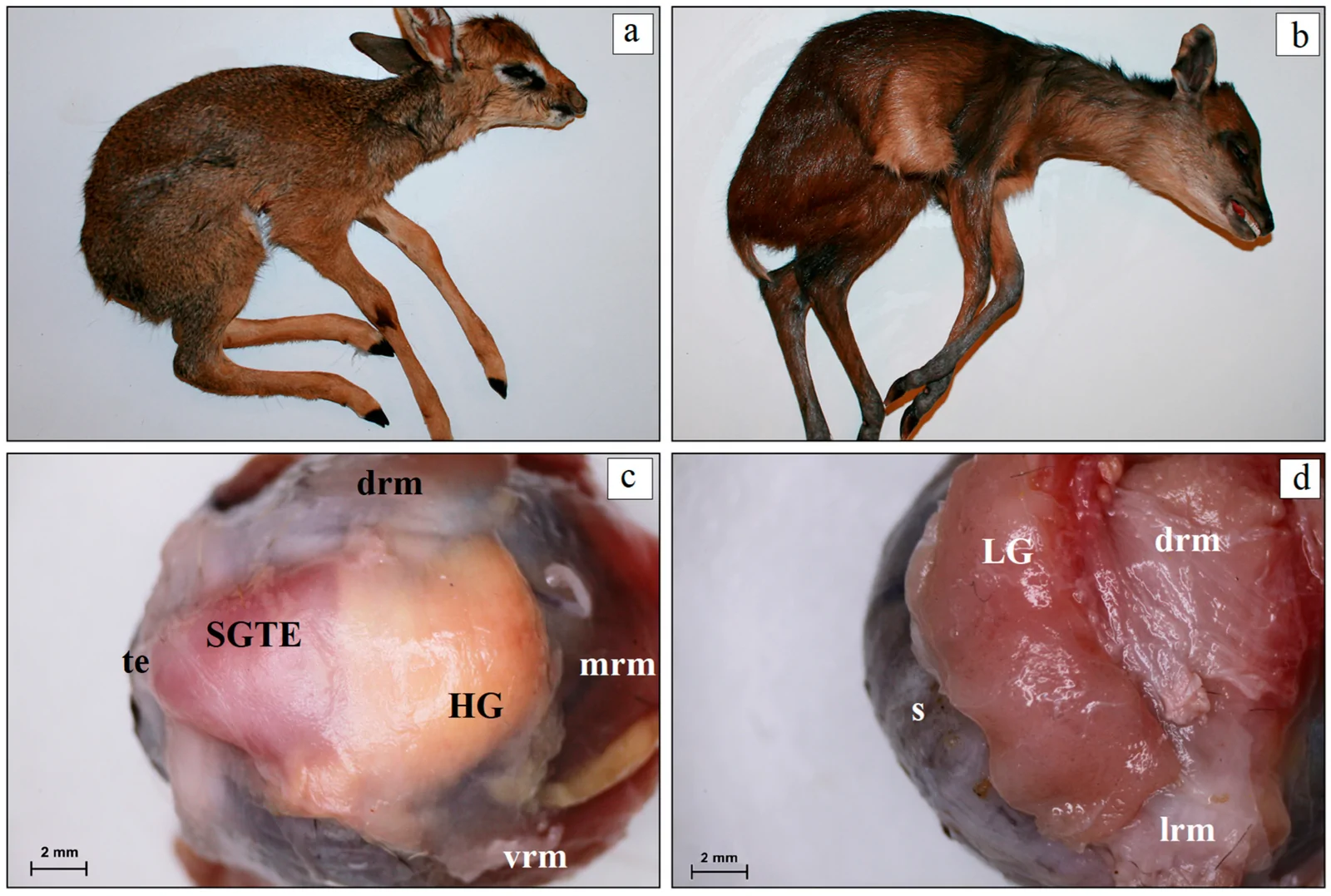
Gross appearance of the examined young wild bovids and gross topography of the orbital glands. (**a**) A young Kirk’s dik-dik (*Madoqua kirkii*). (**b**) A young red forest duiker (*Cephalophorus natalensis*). (**c**) Representative orbital region showing the Harderian gland and the superficial gland of the third eyelid. (**d**) Representative orbital region showing the lacrimal gland and its topographical relationships with the eyeball and extraocular muscles. Abbreviations: drm, dorsal rectus muscle; HG, Harderian gland; LG, lacrimal gland; lrm, lateral rectus muscle; mrm, medial rectus muscle; s, sclera; SGTE, superficial gland of the third eyelid; te, third eyelid; vrm, ventral rectus muscle. Scale bars: (**c**,**d**) 2 mm.

The study included nine young wild ruminants representing two species: four Kirk’s dik-diks (*Madoqua kirkii*) (one male and three females) and five red forest duikers (*Cephalophorus natalensis*; formerly *Cephalophus natalensis*) (three males and two females) (Figure 1a,b). All nine animals were included in the gross anatomical, histological, histometric, and histochemical analyses. In every animal, the HG, SGTE, and LG were available from both sides of the head, and all histological and histochemical staining procedures described in Section 2.4 were applied to each animal. Study codes used in Table 1 were assigned for presentation of the material. The examined species were taxonomically verified according to the Mammal Diversity Database, version 2.4 [25]. Kirk’s dik-dik was classified as *Madoqua kirkii* (A. Günther, 1880), Artiodactyla; Ruminantia; Pecora; Bovidae; Antilopinae; Antilopini; *Madoqua*, with a global IUCN Red List status of Least Concern (LC) (2025-2) [26]. The red forest duiker, also referred to as the Natal red duiker, was classified as *Cephalophorus natalensis* (A. Smith, 1834), Artiodactyla; Ruminantia; Pecora; Bovidae; Antilopinae; Cephalophini; *Cephalophorus*. The global IUCN Red List status of this taxon, assessed under the name *Cephalophus natalensis*, is Least Concern (LC) (2025-2) [26]. The animals originated from the Wrocław Zoological Garden, Poland, where they were maintained under standard zoological husbandry conditions and routine veterinary monitoring. The study was based exclusively on post-mortem material from animals that died independently of the present investigation. The causes of death and general necropsy findings were documented in the zoo’s veterinary records; however, they were outside the scope of the present study and were not used to stratify the material. During tissue collection, no gross abnormalities of the eyeballs or associated orbital tissues were observed. The ocular regions were collected within approximately 2–3 h after death and immediately immersed in 4% buffered formaldehyde (Chempur, Piekary Śląskie, Poland) for at least 72 h. Samples were considered suitable for microscopic analysis when the glandular tissue was intact and the sections showed preserved lobular architecture, identifiable secretory units and ducts, clearly recognizable nuclei and cell boundaries, and no extensive autolysis, tissue fragmentation, necrosis, or diffuse inflammatory destruction. All collected glands met these criteria and were included in the analyses. Because the material was collected post-mortem, functional in vivo assessment of the ocular adnexa was not possible.

### 2.2. Ethical Statement

The study was carried out according to applicable Polish law (Journal of Laws of 15 January 2015—Act on the Protection of Animals Used for Scientific or Educational Purposes) and European regulations (Directive 2010/63/EU of the European Parliament and of the Council of 22 September 2010, on the protection of animals used for scientific purposes). According to both legal acts, studies involving only *post-mortem* material originating from the Wrocław Zoological Garden, Poland, do not require approval from an ethics committee. The material was collected under individual permits (No. PIW Wroc. UT-45/5/16 and No. PIW Wroc. UT-45/6/16) issued by the District Veterinary Officer in Wrocław, Poland.

### 2.3. Macroscopic Examination

After collection, the eyeballs and associated orbital tissues were examined macroscopically. The location, general appearance and topographical relationships of the HG, LG and SGTE were assessed with the naked eye and then using a Zeiss Stemi 2000-C stereomicroscope (Carl Zeiss, Jena, Germany). Holotopy and syntopy were used to describe the position of the examined orbital glands in relation to the eyeball, extraocular muscles and third eyelid. Special attention was paid to the position of each gland within the orbit, its relationship to the rectus and oblique muscles, its association with the third eyelid, its general shape, and the visible course or opening region of the excretory ducts when identifiable. Representative macroscopic photographs were captured with a Canon EOS 300D camera (Canon Inc., Tokyo, Japan). Quantitative macroscopic morphometry of the glands was not performed because priority was given to fixation and preservation of rare tissues for histological and histochemical analyses. Therefore, the gross anatomical results were interpreted as qualitative topographical observations rather than as quantitative size comparisons. Anatomical terminology followed the *Nomina Anatomica Veterinaria* [27].

### 2.4. Histological and Histochemical Stains

Histological and histochemical analyses were performed in all nine animals to describe the microscopic structure of the orbital glands and characterize the chemical classes of their secretory products. Tissue samples were fixed in 4% buffered formaldehyde (Chempur, Piekary Śląskie, Poland) for at least 72 h and then rinsed under running water for 24 h. Dehydration was performed using a graded ethanol series (75%, 96%, and 100%) (Chempur, Piekary Śląskie, Poland) in a vacuum tissue processor (ETP RVG3, Intelsint, Pantigliate, Italy), followed by paraffin embedding. Four-micrometer-thick tissue sections were obtained using a sliding microtome (Slide 2003, Pfm A.G., Cologne, Germany). Mayer’s hematoxylin and eosin staining was used for general histological assessment. Connective tissue components were assessed using Mallory trichrome with aniline blue (Bio-Optica Milano S.p.A., Milan, Italy; cat. no. 04-020802) and modified Russell–Movat pentachrome staining (BioGnost Ltd., Zagreb, Croatia; cat. no. MOV-100T). Methyl green–pyronin Y staining (Bio-Optica Milano S.p.A., Milan, Italy; cat. no. 05-B15003) was used to facilitate the identification of plasma cells. Histochemical analysis included the periodic acid–Schiff reaction (PAS; Bio-Optica Milano S.p.A., Milan, Italy; cat. no. 04-130802) for neutral glycoconjugates; Alcian blue pH 1.0 (AB pH 1.0; Bio-Optica Milano S.p.A., Milan, Italy; cat. no. 04-166802) for strongly sulfated acidic mucosubstances; Alcian blue pH 2.5 (AB pH 2.5; Bio-Optica Milano S.p.A., Milan, Italy; cat. no. 04-160802) for sulfated and carboxylated acidic mucosubstances and sialomucins; combined AB pH 2.5/PAS staining (Bio-Optica Milano S.p.A., Milan, Italy; cat. no. 04-163802), in which acidic components stained blue and neutral PAS-positive components stained magenta; and colloidal iron staining (CI; Bio-Optica Milano S.p.A., Milan, Italy; cat. no. 04-180809) for acidic mucosubstances, including sulfated and carboxylated components. Microscopic imaging was performed using a Zeiss Axio Scope A1 light microscope (Carl Zeiss, Jena, Germany) equipped with AxioVision Release 4.8.2 SP2 software. Histological terminology followed the Nomina Histologica Veterinaria [28]. All procedures involving formaldehyde, xylene, and staining reagents were performed in a fume hood with appropriate personal protective equipment and in accordance with institutional safety regulations.

Histochemical reactions were assessed semiquantitatively by one observer (J.K.-N.) using a procedure modified from Spicer and Henson [29]. Each stained section was first reviewed at low magnification to identify well-preserved secretory parenchyma and exclude folds, tears, and other processing artifacts. Six non-overlapping fields distributed across each section were then evaluated with a 40× objective. The assessment was based on staining intensity in the cytoplasm of secretory epithelial cells and in intraluminal secretory material. Stained area and the proportion of positive secretory units or cells were not scored separately. Each field was assigned one of four primary categories: (−), no specific reaction; (+), weak but clearly detectable reaction; (++), moderate reaction; or (+++), strong reaction. The final score for each section was the predominant category among the six fields. When two adjacent categories occurred with equal frequency, an intermediate score was recorded as (−/+), (+/++), or (++/+++). Right and left glands showed concordant section-level scores in each animal; therefore, a single animal-level score was reported in Table 2. The semiquantitative scores were used only for descriptive comparison and were not subjected to inferential statistical analysis. The assessment was not blinded. Because all scores were assigned by one observer, consensus scoring and interobserver agreement analyses were not applicable.

**Table 2:** Individual histochemical reaction intensities and predominant secretory profiles of the Harderian gland, lacrimal gland, and superficial gland of the third eyelid in young Kirk’s dik-dik and red forest duiker. Kirk’s dik-dik (*Madoqua kirkii*), n = 4 animals (MK-1–MK-4).

Animal ID	Gland	PAS	AB pH 1.0	AB pH 2.5	AB pH 2.5/PAS	CI	Predominant Histochemical Profile
MK -1	**HG**	+++	−/+	+++	blue +++	+++	Mixed profile with a strong acidic component
	**SGTE**	++	+	++	blue +++ (scattered), magenta +++ (predominant)	++	Mixed profile with predominance of neutral PAS-positive components and moderate acidic reactivity
	**LG**	+++	−/+	+++	blue +++ (predominant), magenta +++ (scattered)	+++	Mixed profile with predominant acidic reactivity and a localized neutral PAS-positive component
MK-2	**HG**	++/+++	−/+	+++	blue +++	++/+++	Mixed profile with a strong acidic component
	**SGTE**	+++	+	++	blue +++ (scattered), magenta +++ (predominant)	++	Mixed profile with predominance of neutral PAS-positive components and moderate acidic reactivity
	**LG**	+++	+	+++	blue +++ (predominant), magenta +++ (scattered)	++/+++	Mixed profile with predominant acidic reactivity and a localized neutral PAS-positive component
MK-3	**HG**	+++	+	+++	blue +++	++/+++	Mixed profile with a strong acidic component
	**SGTE**	+++	+	++	blue +++ (scattered), magenta +++ (predominant)	++	Mixed profile with predominance of neutral PAS-positive components and moderate acidic reactivity
	**LG**	+++	+	+++	blue +++ (predominant), magenta +++ (scattered)	+++	Mixed profile with predominant acidic reactivity and a localized neutral PAS-positive component
MK-4	**HG**	+++	−/+	+++	blue +++	+++	Mixed profile with a strong acidic component
	**SGTE**	++	+	++	blue +++ (scattered), magenta +++ (predominant)	++	Mixed profile with predominance of neutral PAS-positive components
	**LG**	+++	−/+	+++	blue +++ (predominant), magenta +++ (scattered)	+++	Mixed profile with predominant acidic reactivity and a localized neutral PAS-positive component

Abbreviations: HG, Harderian gland; LG, lacrimal gland; SGTE, superficial gland of the third eyelid; PAS, periodic acid–Schiff; AB, Alcian blue; CI, colloidal iron. Sample size: Kirk’s dik-dik, n = 4 animals (8 glands of each gland type); red forest duiker, n = 5 animals (10 glands of each gland type). Scoring procedure: Each animal-level score represents the predominant staining intensity recorded in six non-overlapping microscopic fields examined with a 40× objective for each histological preparation. Right and left glands showed concordant staining scores in each animal; therefore, a single animal-level score is reported. Reaction intensity: −/+, very weak; +, weak; ++, moderate; ++/+++, moderate to strong; +++, strong. In AB pH 2.5/PAS staining, blue indicates acidic mucosubstances, whereas magenta indicates neutral PAS-positive components. The terms scattered and predominant describe the qualitative distribution of the two colors and do not represent separate intensity scores.

### 2.5. Histometric Analysis of H&E-Stained Sections

Digital histometric measurements were performed on photomicrographs of H&E-stained sections from all nine animals to provide descriptive quantitative data on the microscopic structure of the HG, SGTE, and LG. For each gland type, one representative H&E-stained section from the right and left sides of every animal was evaluated. Accordingly, eight sections per gland type were examined in Kirk’s dik-dik (4 animals × 2 sides) and ten sections per gland type in the red forest duiker (5 animals × 2 sides), corresponding to 24 and 30 gland sections, respectively, across the three gland types. The selected parameters included capsule thickness, interlobular septal thickness, and the outer diameters of acini and tubules, following El-Fadaly et al. [30], with modifications related to the post-mortem material. Measurements were made only in well-preserved and properly oriented glandular areas. Capsule thickness and interlobular septal thickness were measured in randomly selected regions at 50× magnification. The outer diameters of acini and tubules were measured in central glandular fields at 400× magnification, at the widest diameter of transversely or nearly transversely sectioned profiles. For each section, technical measurements were obtained from selected profiles that fulfilled the preservation and orientation criteria. The number of technical measurements was not fixed a priori and varied according to the amount and orientation of suitable tissue in each section. Measurements were performed using AxioVision Release 4.8.2 SP2 software (Carl Zeiss MicroImaging GmbH, Jena, Germany). For each gland type and examined group, the arithmetic mean was calculated from the pooled technical measurements, and the SD was calculated as the sample standard deviation of those measurements. Because the number of eligible profiles varied among sections, pooled estimates may give unequal weight to individual sections and animals; they were therefore used only as descriptive summaries. The reported SD reflects variability among technical measurements within the examined sections and not biological variability among individual animals. Technical measurements were not treated as independent biological replicates. Because of the small group sizes, no inferential statistical comparisons between species or sexes were performed. The exact total number of technical measurements performed for each parameter was not retained as a separate dataset and could not be reconstructed reliably.

## 3. Results

### 3.1. Gross Topography of the Orbital Gland Complex

In both examined groups, the HG, LG, and SGTE were identified as three topographically distinct components of the orbital gland complex. The HG was oval and located in the medioventral region of the orbit, on the ventral rectus muscle. The gland had anterior and posterior extremities and dorsal and ventral borders. Its posterior extremity was directed toward the orbital apex and was related to the ventral border of the SGTE and the ventral oblique muscle (Figure 1c). A single excretory duct was observed on the medial surface of the gland. This position placed the HG in close anatomical association with the medial conjunctival region and the third eyelid. The SGTE was oval, light pink, and located in the medial part of the orbit, between the medial and ventral rectus muscles. It was partially covered by the ventral oblique muscle and appeared uniform and undivided (Figure 1c). Its medial position and close relationship with the third eyelid separated it from the more ventrally located HG. By contrast, the LG was flat, triangular in outline, and light pink. It was located in the dorsolateral part of the orbit, between the dorsal and lateral rectus muscles. The LG drained through small excretory ductules that opened into the upper conjunctival fornix (Figure 1d). Thus, the three glands formed a distinct medioventral–medial–dorsolateral arrangement within the orbit, with the HG associated mainly with the ventral orbital region, the SGTE with the third eyelid, and the LG with the upper conjunctival fornix. The main gross topographical features and anatomical relationships are presented separately for the two examined groups in Table 3.

### 3.2. Histological Study

No consistent sex-related pattern was apparent in the microscopic organization of the examined glands. Males and females showed the same general arrangement of secretory units, ducts, and connective tissue components, but the small numbers did not permit formal evaluation of sex-related variation. Histometric values were therefore presented jointly for both sexes without inferential statistical analysis. The microscopic description below focuses on capsule and septal development, lobular arrangement, acinar and tubular components, myoepithelial cells, and the distribution of plasma cells and lymphocytes.

#### 3.2.1. Kirk’s Dik-Dik

**Figure 2:**
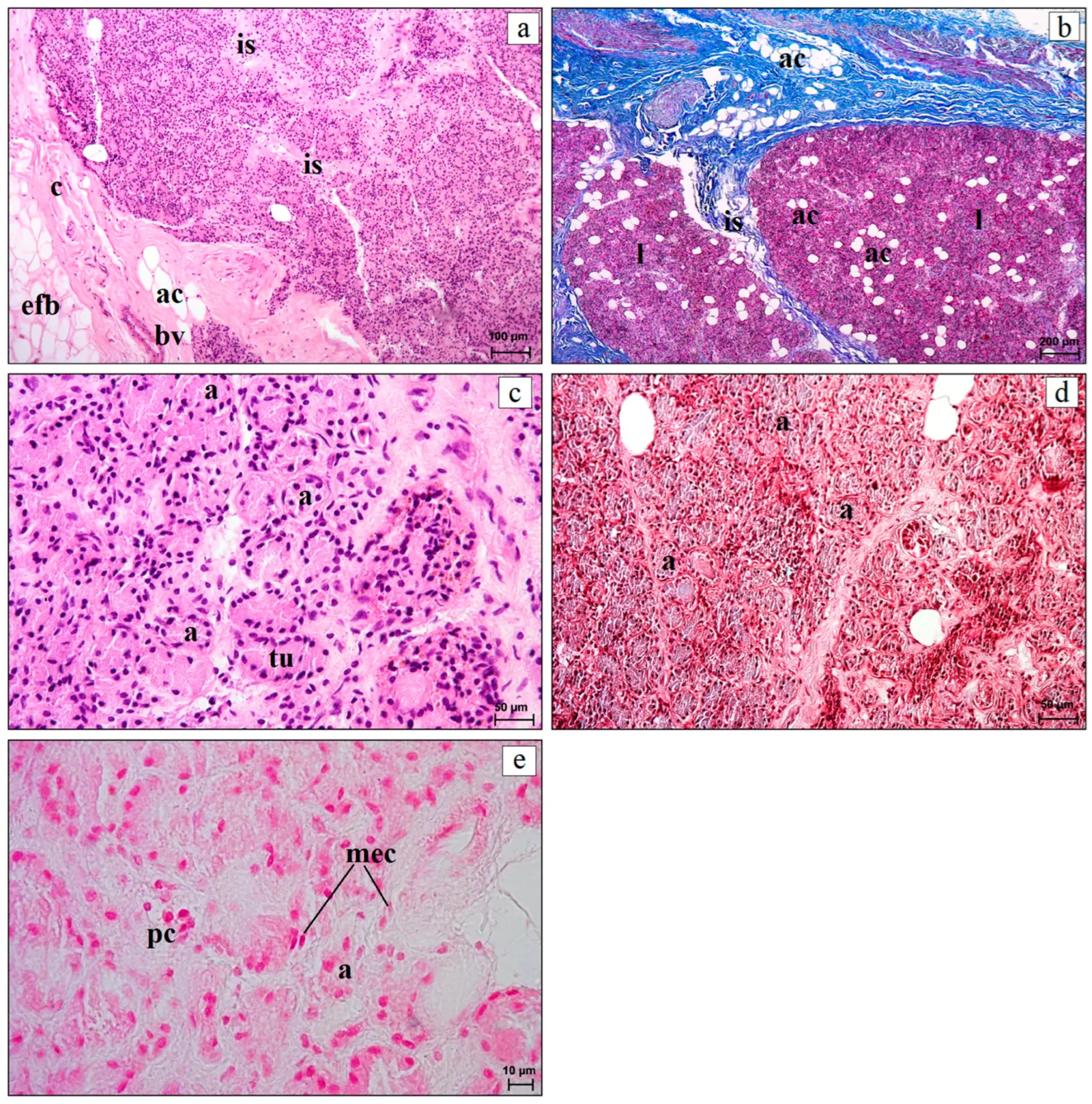
Representative histological features of the Harderian gland in young Kirk’s dik-diks (*Madoqua kirkii)*. (**a**) General lobular organization of the gland, with a thick connective tissue capsule and an associated extraperiorbital fat body. (**b**) Connective tissue capsule and interlobular septa dividing the glandular parenchyma into lobes; adipocytes are visible within the connective tissue. (**c**) Tubuloacinar organization, showing acini with narrow lumina and tubules with wider lumina. (**d**) Modified Russell–Movat pentachrome staining showing a weak reaction in selected secretory units. (**e**) High-magnification view of myoepithelial cells and scattered plasma cells in the glandular interstitium. Abbreviations: a, acini; ac, adipocytes; bv, blood vessels; c, capsule; efb, extraperiorbital fat body; is, interlobular septa; l, lobes; mec, myoepithelial cells; pc, plasma cells; tu, tubules. Staining: (**a**,**c**) Mayer’s hematoxylin and eosin; (**b**) Mallory trichrome with aniline blue; (**d**) modified Russell–Movat pentachrome; (**e**) methyl green–pyronin Y. Scale bars: (**a**) 100 µm; (**b**) 200 µm; (**c**,**d**) 50 µm; (**e**) 10 µm.

In the examined Kirk’s dik-diks, the HG was a compound tubuloacinar gland surrounded by a thick connective tissue capsule composed of collagen, reticular and elastic fibers, blood vessels, fibrocytes, and numerous adipocytes (Figure 2a,b). The gland was also associated with an extraperiorbital fat body (Figure 2a). Interlobular connective tissue septa divided the gland into clearly visible large and small lobes (Figure 2a,b). Numerous adipocytes were also present in the glandular interstitium (Figure 2b). The acini had small lumina and were lined by tall conical cells with eosinophilic cytoplasm and large, round, basally located nuclei (Figure 2c). The tubules had large lumina and were lined by a single layer of cuboidal cells (Figure 2c). Myoepithelial cells surrounded the acini (Figure 2e). Modified Russell–Movat pentachrome staining showed a very weak positive reaction in the acini, assessed as −/+ (Figure 2d). Methyl green–pyronin Y staining showed a few plasma cells in the glandular interstitium (Figure 2e).

**Figure 3:**
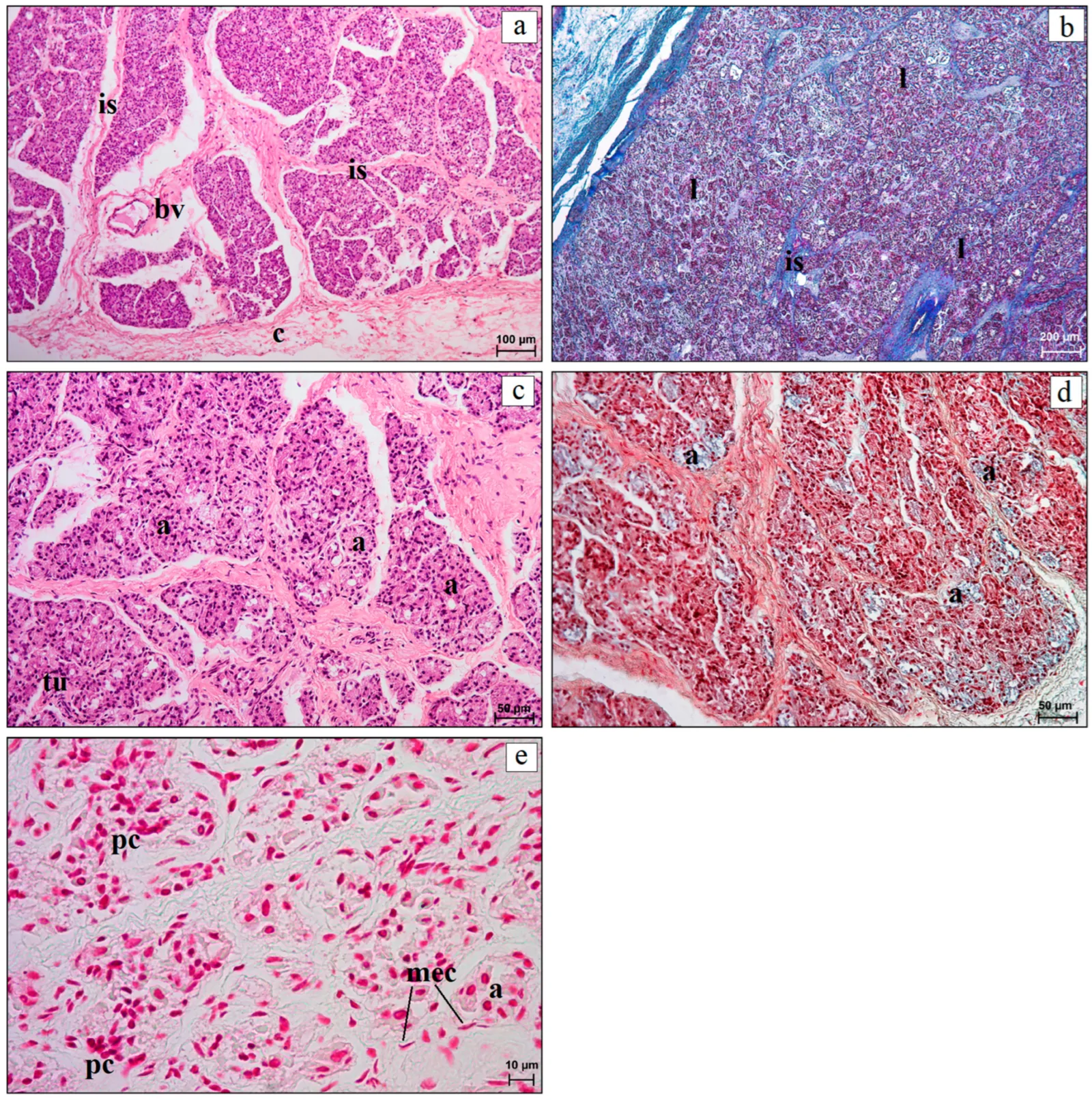
Representative histological features of the superficial gland of the third eyelid in young Kirk’s dik-diks (*Madoqua kirkii*). (**a**) General organization of the gland, with a thick connective tissue capsule and a lobular arrangement of the parenchyma. (**b**) Capsule and interlobular septa composed mainly of connective tissue and containing blood vessels. (**c**) Predominantly acinar secretory parenchyma, with acini surrounded by basal myoepithelial cells. (**d**) Modified Russell–Movat pentachrome staining showing mucosubstance-containing secretory units. (**e**) High-magnification view of myoepithelial cells and plasma cells within the glandular interstitium. Abbreviations: a, acini; bv, blood vessels; c, capsule; is, interlobular septa; l, lobes; mec, myoepithelial cells; pc, plasma cells; tu, tubules. Staining: (**a**,**c**) Mayer’s hematoxylin and eosin; (**b**) Mallory trichrome with aniline blue; (**d**) modified Russell–Movat pentachrome; (**e**) methyl green–pyronin Y. Scale bars: (**a**) 100 µm; (**b**) 200 µm; (**c**,**d**) 50 µm; (**e**) 10 µm.

The SGTE was a compound tubuloacinar gland with a predominance of acini. It was surrounded by a thick connective tissue capsule composed of collagen, reticular, and elastic fibers, blood vessels, and fibrocytes (Figure 3a,b). Connective tissue extended from the capsule into the glandular parenchyma and formed thin interlobular septa that divided the gland into large lobes (Figure 3a,b). The acini had small lumina and were composed of tall conical cells surrounded by basal myoepithelial cells (Figure 3c,e). The secretory cells had eosinophilic granular cytoplasm (Figure 3c). The tubules had large lumina and were lined by a single layer of cuboidal cells. Modified Russell–Movat pentachrome staining showed numerous secretory units containing mucous secretion, with a reaction assessed as +/++ (Figure 3d). Methyl green–pyronin Y staining showed numerous plasma cells in the glandular interstitium (Figure 3e).

**Figure 4:**
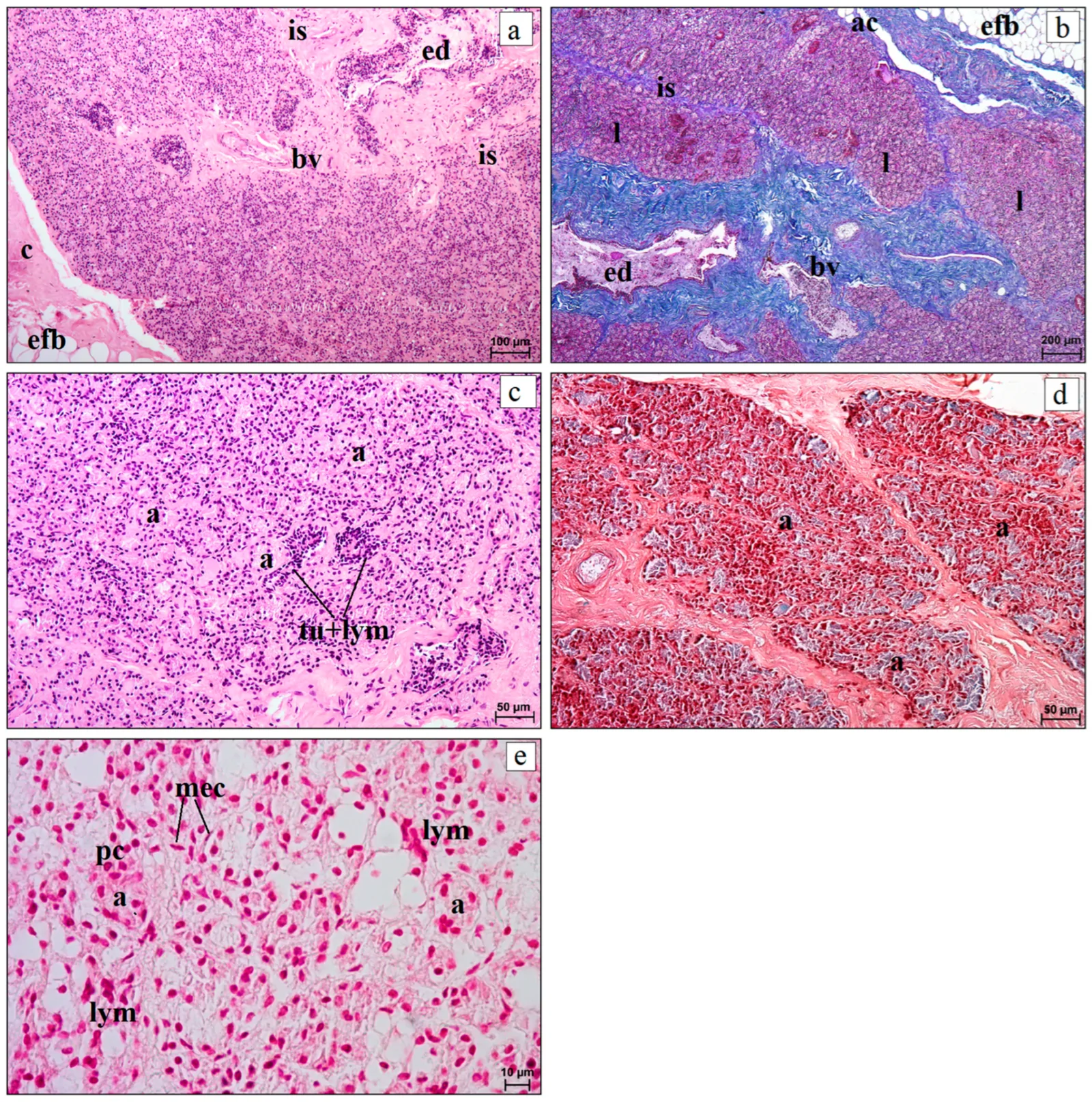
Representative histological features of the lacrimal gland in young Kirk’s dik-diks (*Madoqua kirkii*). (**a**) General lobular organization of the gland, with a connective tissue capsule and associated extraperiorbital fat body. (**b**) Capsule and interlobular septa dividing the parenchyma into poorly demarcated lobes; adipocytes and blood vessels are present within the connective tissue. (**c**) Tubuloacinar organization with acini, tubules, and small lymphocyte aggregates adjacent to tubular profiles. (**d**) Modified Russell–Movat pentachrome staining showing mucosubstance-containing secretory units. (**e**) High-magnification view of myoepithelial cells, scattered plasma cells, and lymphocytes in the glandular interstitium. Abbreviations: a, acini; ac, adipocytes; bv, blood vessels; c, capsule; efb, extraperiorbital fat body; is, interlobular septa; l, lobes; lym, lymphocytes; mec, myoepithelial cells; pc, plasma cells; tu, tubules. Staining: (**a**,**c**) Mayer’s hematoxylin and eosin; (**b**) Mallory trichrome with aniline blue; (**d**) modified Russell–Movat pentachrome; (**e**) methyl green–pyronin Y. Scale bars: (**a**) 100 µm; (**b**) 200 µm; (**c**,**d**) 50 µm; (**e**) 10 µm.

The LG was a compound tubuloacinar gland with a predominance of acini. It was surrounded by a thick connective tissue capsule composed of collagen, reticular, and elastic fibers, fibrocytes, numerous blood vessels, and occasional adipocytes (Figure 4a,b). The capsule was surrounded by extraperiorbital adipose tissue (Figure 4a,b). Thin connective tissue septa extended from the capsule into the glandular parenchyma and divided the gland into poorly demarcated large lobes (Figure 4b). The acini had small lumina and were composed of tall conical cells surrounded by myoepithelial cells. The acinar cells had eosinophilic granular cytoplasm (Figure 4c,e). The tubules had large lumina and were lined by a single layer of cuboidal cells (Figure 4c). Small lymphocytic aggregates were observed around selected tubules (Figure 4c). Modified Russell–Movat pentachrome staining showed numerous secretory units containing mucous secretion, with a reaction assessed as ++ (Figure 4d). Scattered plasma cells and numerous lymphocytes were present in the glandular interstitium (Figure 4e).

Among the examined Kirk’s dik-diks, the three glands shared a compound tubuloacinar organization but differed in stromal development and secretory-unit appearance. The HG contained numerous adipocytes in the capsule and interstitium, whereas the SGTE and LG contained more evident mucous material in modified Russell–Movat pentachrome preparations. These observations describe the available specimens and were not tested as species-level differences.

#### 3.2.2. Red Forest Duiker

In the examined red forest duikers, the HG was a compound tubuloacinar gland surrounded by a very thick connective tissue capsule (Figure S1a). The capsule contained collagen, reticular and elastic fibers, fibrocytes, numerous blood vessels, and clusters of adipocytes (Figure S1a,b). Thick interlobular connective tissue septa divided the glandular parenchyma into small lobes (Figure S1b). The acini were composed of tall conical secretory cells with basophilic cytoplasm and were surrounded by myoepithelial cells (Figure S1c,e). The tubules had large lumina and were lined by a single layer of cuboidal cells (Figure S1c). Modified Russell–Movat pentachrome staining showed a positive reaction in a small number of acini containing mucous secretion, assessed as ++ (Figure S1d). Numerous clusters of plasma cells were present in the glandular interstitium (Figure S1e).

In the examined red forest duikers, the SGTE was a compound tubuloacinar gland with a predominance of acini. It was surrounded by a thick connective tissue capsule composed of collagen, reticular and elastic fibers and fibrocytes (Figure S2a). Thick interlobular connective tissue septa divided the gland into small and medium-sized lobes (Figure S2b). The acini had small lumina and were composed of tall conical cells surrounded by basal myoepithelial cells. The glandular cells had eosinophilic granular cytoplasm (Figure S2c,e). The tubules had large lumina and were lined by a single layer of cuboidal cells (Figure S2c). Modified Russell–Movat pentachrome staining showed numerous acini containing mucous secretion, with a reaction assessed as ++/+++ (Figure S2d). A few plasma cells and numerous lymphocytes were present in the glandular interstitium (Figure S2e).

In the examined red forest duikers, the LG was a compound tubuloacinar gland surrounded by a thick connective tissue capsule composed of collagen, reticular and elastic fibers and fibrocytes (Figure S3a). No adipose tissue was observed within the capsule or interlobular connective tissue. Thick interlobular septa containing numerous blood vessels extended from the capsule into the glandular parenchyma and divided it into large lobes (Figure S3b). The lobes contained branched tubular secretory units with demilune-like acinar terminal portions (Figure S3c). The acinar secretory cells had basophilic cytoplasm (Figure S3c). The tubules were lined by a single layer of cuboidal cells with large, oval, basally located nuclei. Modified Russell–Movat pentachrome staining showed very numerous acini containing mucous secretion, with a reaction assessed as +++ (Figure S3d). Scattered plasma cells were present in the glandular interstitium (Figure S3e).

Among the examined red forest duikers, the HG had the most prominent connective tissue capsule, whereas the LG showed branched tubular units with acinar terminal portions. Modified Russell–Movat pentachrome staining was strongest in the LG, indicating a more evident mucous component in this gland. These findings are reported as descriptive features of the examined group.

### 3.3. Descriptive Histometry of the Orbital Glands

**Figure 5:**
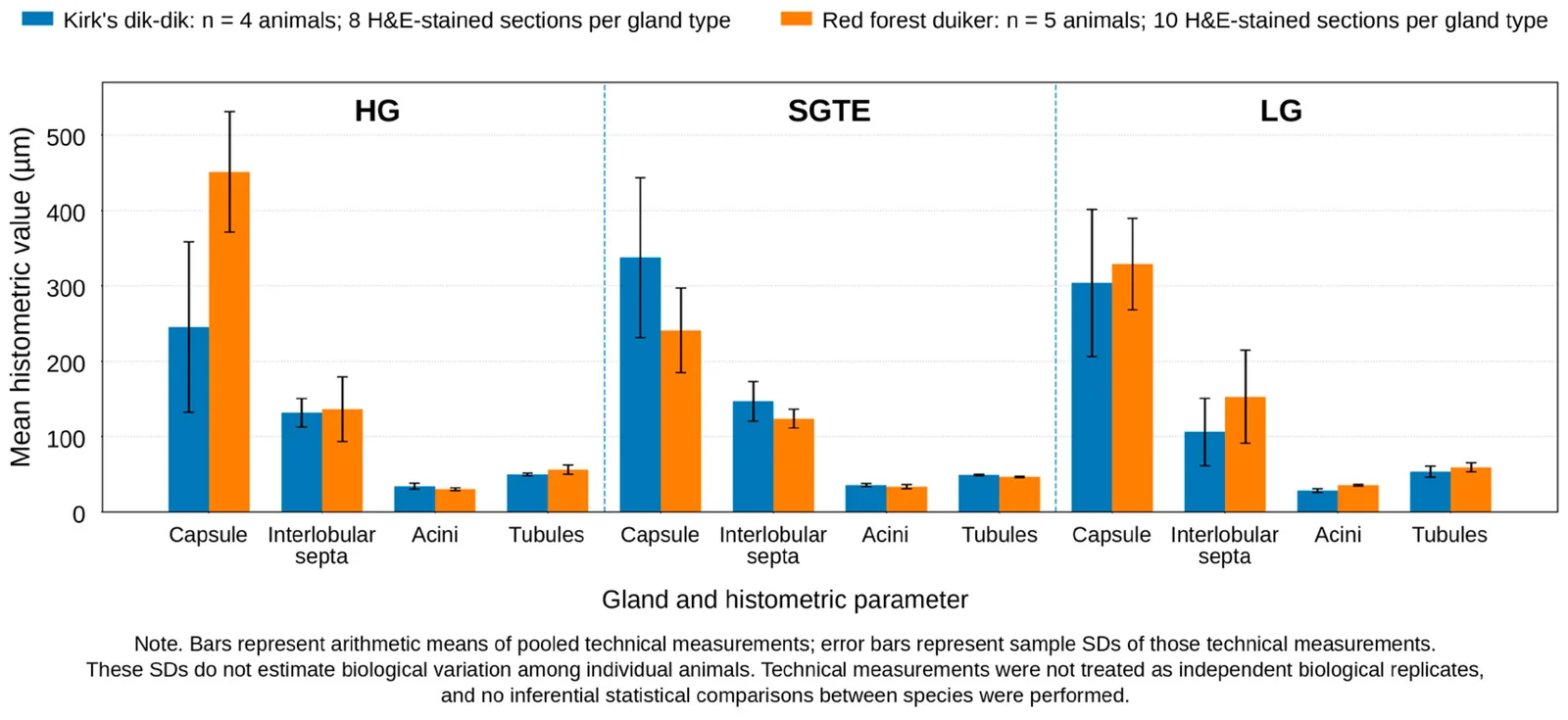
Descriptive histometric measurements of selected parameters of the Harderian gland (HG), superficial gland of the third eyelid (SGTE), and lacrimal gland (LG) in Kirk’s dik-dik (n = 4 animals; 8 H&E-stained sections for each gland type) and red forest duiker (n = 5 animals; 10 H&E-stained sections for each gland type). One H&E-stained section from each right and left gland was evaluated. The graph presents the mean capsule thickness, interlobular septal thickness, and outer diameters of acini and tubules. Bars represent arithmetic means calculated from pooled technical measurements obtained from the indicated sections, and error bars represent the sample standard deviations of those technical measurements. Because the standard deviations were not calculated from animal-level means, they do not estimate biological variation among individual animals. Technical measurements were not treated as independent biological replicates, and no inferential statistical comparisons between species were performed. The exact number of technical measurements performed for each parameter was not retained as a separate dataset and could not be reconstructed reliably.

Histometric evaluation showed descriptive variation between the two examined groups (Figure 5). Measurements from all nine animals were summarized without inferential statistical testing; therefore, the numerical differences reported below indicate patterns in the available material rather than statistically established differences between species. For each gland type, values for Kirk’s dik-dik were derived from eight bilateral H&E-stained sections and those for the red forest duiker from ten bilateral H&E-stained sections. Means and SDs were calculated from pooled technical measurements obtained from these sections. The technical measurements were not independent biological replicates, and the reported SD represents variability among technical measurements rather than biological variation among individual animals.

In the HG, the largest numerical difference concerned connective tissue capsule thickness. The pooled mean capsule thickness was numerically higher in the red forest duiker group (451.04 ± 79.90 µm) than in the Kirk’s dik-dik group (245.37 ± 113.06 µm). In contrast, the pooled mean thickness of the interlobular septa was broadly comparable between the groups, measuring 136.09 ± 42.90 µm in the red forest duiker group and 131.59 ± 18.85 µm in the Kirk’s dik-dik group. The pooled mean outer diameter of the HG acini was slightly higher in the Kirk’s dik-dik group (33.97 ± 4.10 µm) than in the red forest duiker group (29.76 ± 1.90 µm), whereas the pooled mean outer diameter of the tubules was higher in the red forest duiker group (55.87 ± 6.10 µm) than in the Kirk’s dik-dik group (49.72 ± 1.70 µm).

In the SGTE, the Kirk’s dik-dik group had numerically higher pooled mean values for all measured parameters. The capsule measured 337.54 ± 106.06 µm in the Kirk’s dik-dik group and 240.77 ± 56.20 µm in the red forest duiker group. The interlobular septa measured 146.60 ± 26.20 µm and 123.75 ± 12.30 µm, respectively. The pooled mean outer diameter of the acini was 35.61 ± 2.01 µm in the Kirk’s dik-dik group and 33.33 ± 3.10 µm in the red forest duiker group, whereas the pooled mean outer diameter of the tubules was 48.92 ± 0.90 µm and 46.34 ± 0.90 µm, respectively.

In the LG, most pooled mean histometric parameters were numerically higher in the red forest duiker group. Capsule thickness was 329.01 ± 60.70 µm in the red forest duiker group and 303.84 ± 97.70 µm in the Kirk’s dik-dik group. The pooled mean thickness of the interlobular septa was also higher in the red forest duiker group (152.69 ± 61.70 µm) than in the Kirk’s dik-dik group (105.90 ± 44.90 µm). The pooled mean outer diameter of the acini was 35.48 ± 1.20 µm in the red forest duiker group and 27.93 ± 2.40 µm in the Kirk’s dik-dik group, whereas the pooled mean outer diameter of the tubules was 58.85 ± 5.90 µm and 53.29 ± 7.30 µm, respectively.

The largest numerical difference in the pooled measurements concerned the HG capsule, for which the mean was higher in the red forest duiker group, whereas the Kirk’s dik-dik group had higher pooled SGTE measurements. These values are descriptive technical summaries and should be confirmed in larger studies using animal-level measurements and independently sampled groups.

### 3.4. Histochemical Secretory Profiles

The histochemical characteristics of the HG, LG, and SGTE in the examined young wild ruminants are summarized in Table 2 and illustrated in Supplementary Figures S4–S9. The staining patterns showed variation among gland types and between the two examined groups. All glands contained both neutral PAS-positive components and acidic mucosubstances, although their relative proportions varied.

Individual histochemical scores showed within-group variation (Table 2). Among Kirk’s dik-diks, the HG showed PAS reactions ranging from moderate-to-strong to strong (++/+++ to +++), AB pH 1.0 reactions from very weak to weak (−/+ to +), consistently strong AB pH 2.5 and blue AB pH 2.5/PAS reactions (+++), and CI reactions ranging from moderate-to-strong to strong (++/+++ to +++). The SGTE showed PAS reactions from moderate to strong (++ to +++), consistently weak AB pH 1.0 reactions (+), moderate AB pH 2.5 and CI reactions (++), and combined AB pH 2.5/PAS staining with scattered blue and predominant magenta components. The LG showed consistently strong PAS and AB pH 2.5 reactions (+++), very weak to weak AB pH 1.0 reactions (−/+ to +), CI reactions ranging from moderate-to-strong to strong (++/+++ to +++), and combined staining with a predominant blue component and scattered magenta staining. Among red forest duikers, the HG showed PAS reactions from moderate to strong (++ to +++), AB pH 1.0 reactions from very weak to weak (−/+ to +), AB pH 2.5 reactions from moderate-to-strong to strong (++/+++ to +++), CI reactions from moderate to strong (++ to +++), and combined staining with predominant magenta and scattered blue components. The SGTE showed PAS reactions from moderate-to-strong to strong (++/+++ to +++), negative to very weak AB pH 1.0 reactions (− to −/+), consistently moderate AB pH 2.5 reactions (++), CI reactions from moderate to strong (++ to +++), and magenta-only combined reactions ranging from moderate to strong (++ to +++). The LG showed PAS reactions from moderate to strong (++ to +++), consistently weak AB pH 1.0 reactions (+), AB pH 2.5 and CI reactions from moderate to strong (++ to +++), and combined staining with predominant magenta and scattered blue components.

Overall, the histochemical results showed mixed secretory profiles in both examined groups, with neutral glycoconjugates and acidic mucosubstances present in the HG, SGTE, and LG. Weak or absent AB pH 1.0 reactions indicated that strongly sulfated acidic mucosubstances were limited. Positive AB pH 2.5 and CI reactions were consistent with the presence of carboxylated and/or weakly sulfated acidic components, although these classical stains did not permit precise molecular identification. Variation in the proportions of blue and magenta staining in AB pH 2.5/PAS preparations reflected differences in the balance of acidic and neutral secretory material among glands, animals, and the two examined groups.

## 4. Discussion

This preliminary descriptive study documented the bilateral HG, SGTE, and LG in all nine examined young bovids. The gross arrangement was consistent in the available material: the HG occupied the medioventral orbit, the SGTE lay medially in close relation to the third eyelid, and the LG was located dorsolaterally. Comparable relationships between the orbital glands, extraocular muscles, conjunctival fornices, and third eyelid have been reported in cattle, American bison, camelids, and several wild ruminants [7,9,15,17,18,19,23,24]. The medioventral position of the HG and the medial position of the SGTE allowed these glands to be distinguished during dissection, despite their proximity to the third eyelid and ventral extraocular muscles. The LG was clearly separated by its dorsolateral position and relationship with the upper conjunctival fornix. These observations are useful for identifying normal glandular tissue during post-mortem examination and for distinguishing the three components of the orbital gland complex. They do not provide direct evidence of glandular function because tear production, secretory activity, and ocular surface physiology were not evaluated in vivo.

The presence of all three glands warrants comparison with other non-domestic ruminants. Klećkowska-Nawrot et al. [31] reported variation in the orbital glandular apparatus of okapi, Père David’s deer, and Philippine mouse-deer, including the absence of an identifiable HG in the examined okapi. Rehorek et al. [7] proposed that the HG and SGTE of deer may represent deep and superficial lobes of one anterior orbital gland. In the current material, the HG and SGTE were described as separate structures according to classical veterinary anatomical terminology because distinct glandular masses and topographic relationships could be recognized. Their close association in the medial orbital region nevertheless supports careful interpretation when these structures are compared among ruminants. Differences in terminology, dissection approach, preservation, and the degree of continuity between glandular masses may partly explain contrasting descriptions in the literature. Given the small numbers examined here, the recorded presence and arrangement of the glands should be regarded as preliminary observations rather than a complete characterization of either species.

The HG in both groups had a compound tubuloacinar organization supported by a connective tissue capsule and interlobular septa. This general pattern agrees with descriptions in camel, buffalo, roe deer, red deer, American bison, cattle, sheep, goats, alpaca, European bison, Père David’s deer, and Philippine mouse-deer [9,15,17,18,19,20,31,32,33,34]. The largest descriptive difference in the pooled technical measurements was the higher HG capsule thickness in the red forest duiker group. In the Kirk’s dik-dik group, adipocytes were particularly evident in the capsule and glandular interstitium. A capsule containing collagen, reticular, and elastic fibers provides the structural framework of the gland, while septa support vessels and divide the parenchyma into lobes. The degree of capsule and septal development should therefore be recorded when reference and altered orbital tissues are compared. However, capsule thickness and adipose tissue distribution may vary with individual anatomy, age, tissue orientation, fixation, or other biological factors. The present findings do not establish these features as species-specific or adaptive.

Histometric measurements supported several microscopic observations, including the relatively high pooled mean HG capsule thickness in the red forest duiker group and the higher pooled SGTE measurements in the Kirk’s dik-dik group. Acinar and tubular diameters also differed numerically between glands and groups. These values were derived from bilateral sections from all animals, but the small group sizes did not justify inferential statistical testing. In addition, capsule and septal measurements can be affected by the plane of section and local variation within a gland, while acinar and tubular diameters depend on the orientation of individual secretory units. The use of repeated technical measurements improves the description of the selected sections but does not increase the number of independent animals. Because the number of eligible profiles varied among sections, pooling technical measurements may also give unequal weight to individual sections and animals. For these reasons, the reported values summarize technical measurements from the available material and should not be interpreted as population estimates. Future work should use predefined sampling regions, a fixed number of measurements, animal-level averages, and larger groups to determine whether the numerical patterns are reproducible.

The SGTE was compound tubuloacinar with a predominance of acini in both groups. Its capsule, interlobular septa, and lobular arrangement were comparable to those described in domestic mammals and other ruminants [1,2,7,15,18,19]. The pooled SGTE measurements were numerically higher in the Kirk’s dik-dik group, whereas the red forest duikers had thick septa dividing the gland into small and medium-sized lobes. These patterns show variation in stromal organization within the examined material, but their consistency at the species level remains unknown. The close relationship of the SGTE to the third eyelid agrees with the established anatomy of the nictitans gland. Nevertheless, the present histological and histochemical observations cannot determine the volume, composition, or rate of secretion reaching the conjunctival sac.

The LG also fell within the known structural range of mammalian lacrimal glands, which may contain acinar, tubular, or tubuloacinar components [17,19,20]. In the examined red forest duikers, the LG contained branched tubular units with demilune-like acinar terminal portions. This designation was based solely on their conventional light-microscopic appearance. Because no ultrastructural, immunohistochemical, or secretion-specific criteria were applied, these profiles should not be interpreted as confirmed serous demilunes or assigned a specific functional role. The Kirk’s dik-dik LG was predominantly tubuloacinar and had less clearly demarcated lobes. Comparable variation in lacrimal gland architecture and staining properties has been reported among American bison, cattle, alpaca, European bison, and selected wild ruminants [9,15,18,19,31]. The observed arrangement of secretory units may reflect differences in the cellular composition of the glands, but this cannot be resolved by routine light microscopy alone. Ultrastructural examination and epithelial markers would be required before these features could be linked to distinct secretory cell populations or physiological properties.

Plasma cells and lymphocytes were present in the glandular interstitium. Similar cell populations have been described in the conjunctiva, third eyelid, and orbital glands of domestic and wild ruminants [15,31,35,36]. In the current sections, the cells were scattered or formed small aggregates, but organized lymphoid follicles were not identified. Their presence may form part of the expected microscopic background of the orbital glands and is relevant when post-mortem tissues are assessed for inflammation. This interpretation must remain limited because plasma cells were identified by morphology and methyl green–pyronin Y staining, and no immunohistochemical markers were used. The observations document immune-cell populations in the examined glands but do not demonstrate their activity, immunoglobulin class, or contribution to ocular surface defense.

The histochemical reactions demonstrated mixed secretory profiles containing neutral glycoconjugates and acidic mucosubstances. PAS, AB pH 2.5, AB pH 2.5/PAS, and CI reactions varied among the HG, SGTE, and LG, among individual animals, and between the two groups. Similar combinations of PAS-positive and Alcian blue-positive material have been reported in ruminant orbital glands [9,15,17,18,19,23,24,31,34]. Weak or absent AB pH 1.0 reactions suggested that strongly sulfated mucosubstances were limited, whereas positive AB pH 2.5 and CI reactions were consistent with a greater contribution of carboxylated and/or weakly sulfated acidic components. The combined AB pH 2.5/PAS preparations showed that the balance of acidic and neutral material differed among glands. The semiquantitative scores were based on six fields per section and one observer. This standardized procedure allowed the staining methods to be assessed descriptively, but interobserver agreement and variation outside the selected fields were not evaluated. Classical histochemistry identifies broad chemical classes within fixed tissue but does not measure the molecular composition, concentration, volume, or physical properties of the secreted tear film. The staining patterns may be discussed in relation to the established protective roles of orbital gland secretions, but they do not prove differences in lubrication, tear-film stability, or other physiological functions.

The principal value of the study is the documentation of gross, histological, histometric, and histochemical features in rarely examined zoo-maintained bovids. Such information may assist comparative anatomists and veterinary pathologists when identifying orbital gland tissue or interpreting structural changes in post-mortem specimens. The observations may also help recognize the reference appearance of stromal, epithelial, and immune-cell components, although clinical interpretation requires appropriate controls and case history. The study remains limited by the small numbers of animals, the absence of exact age data, the restriction to young zoo-maintained individuals, and the lack of in vivo functional, biochemical, immunohistochemical, and ultrastructural analyses. Because all animals were young and maintained in one zoological collection, age, husbandry, and taxon-related effects cannot be separated. Gross gland dimensions were not recorded before fixation, and the histometric results were descriptive technical summaries. Larger series with balanced age and sex groups are required to determine whether the recorded patterns are consistent within each species and whether they differ from those of other bovids.

## 5. Conclusions

This preliminary descriptive study documented the gross topography, microscopic organization, histometric features, and histochemical reactions of the HG, SGTE, and LG in four young Kirk’s dik-diks and five young red forest duikers. All three glands were present bilaterally in every animal and occupied distinct medioventral, medial, and dorsolateral orbital regions. The examined material showed variation in capsule and septal development, lobular arrangement, secretory-unit morphology, and the balance of neutral and acidic secretory components. Plasma cells and lymphocytes were also present in the glandular interstitium. These observations provide reference data for the identification and microscopic evaluation of orbital glands in rarely examined wild bovids. They do not demonstrate functional specialization or establish definitive differences between species. Larger studies using standardized morphometry, immunohistochemistry, ultrastructural methods, and functional assessment are needed to determine whether the recorded patterns are consistent within each species. The main limitations were the opportunistic post-mortem origin of the material and the small group sizes. All nine animals and all bilateral HG, SGTE, and LG samples were included in gross anatomical, histological, histometric, and histochemical analyses, but four Kirk’s dik-diks and five red forest duikers are insufficient for definitive species-level characterization or inferential comparisons. The sex of each animal was recorded, although the group sizes did not permit reliable evaluation of sex-related variation. Exact ages were unavailable, and all animals were classified only as young; age-related and developmental effects could therefore not be assessed. The study was restricted to zoo-maintained animals, and no in vivo measurements of tear production, ocular surface physiology, or glandular secretion were possible. Quantitative gross morphometry was not performed because preservation of the rare tissues for microscopy was prioritized. Histometric data were descriptive technical summaries. The number of eligible profiles varied among sections, so pooled means may have given unequal weight to individual sections and animals, and the reported SDs do not represent biological variation among animals. Classical histochemical staining identified broad classes of secretory material without biochemical confirmation. Cell identification was based on routine morphology and methyl green–pyronin Y staining; immunohistochemical, ultrastructural, and molecular methods were not used. Although causes of death and general necropsy findings were documented in the zoo’s veterinary records, they were not analyzed as study variables. Consequently, a possible influence of terminal systemic condition on glandular morphology or histochemical reactivity cannot be excluded. This risk was reduced by collection within approximately 2–3 h after death and by excluding tissues with extensive autolysis, fragmentation, necrosis, or diffuse inflammatory destruction. The semiquantitative histochemical assessment was performed by one non-blinded observer, and interobserver reproducibility was not evaluated. The absence of a separately retained record of the exact number of technical measurements for each histometric parameter is an additional limitation. Future studies should examine larger series of Kirk’s dik-diks, red forest duikers, and other small wild bovids, with balanced representation of males and females and, when possible, animals of known age. Standardized collection and fixation protocols would reduce preservation bias and permit more reliable morphometric comparisons. Gross gland dimensions and photographic documentation should be obtained before fixation whenever tissue condition allows. Immunohistochemical and ultrastructural methods could further characterize secretory epithelial cells, myoepithelial cells, immune cells, vascular structures, extracellular matrix components, and secretory granules. Histochemical observations should also be compared with biochemical analysis of glandular secretion or the precorneal tear film. In zoo-maintained animals, integration with ophthalmic examination, tear-film testing, and clinical records would help determine whether the structural patterns observed in post-mortem material have consistent physiological or clinical associations.

## Figures and Tables

**Table 1:** Basic information on the animals and analyses performed.

Code	Species	Sex	Age	Glands (Bilateral)	Gross	Histology/ Histometry	Histochemistry
MK-1	Kirk’s dik-dik	M	Young	HG, SGTE, LG	Yes	Yes	All stains
MK-2	Kirk’s dik-dik	F	Young	HG, SGTE, LG	Yes	Yes	All stains
MK-3	Kirk’s dik-dik	F	Young	HG, SGTE, LG	Yes	Yes	All stains
MK-4	Kirk’s dik-dik	F	Young	HG, SGTE, LG	Yes	Yes	All stains
CN-1	Red forest duiker	M	Young	HG, SGTE, LG	Yes	Yes	All stains
CN-2	Red forest duiker	M	Young	HG, SGTE, LG	Yes	Yes	All stains
CN-3	Red forest duiker	M	Young	HG, SGTE, LG	Yes	Yes	All stains
CN-4	Red forest duiker	F	Young	HG, SGTE, LG	Yes	Yes	All stains
CN-5	Red forest duiker	F	Young	HG, SGTE, LG	Yes	Yes	All stains

HG, Harderian gland; SGTE, superficial gland of the third eyelid; LG, lacrimal gland. All three glands were available bilaterally in every animal. Gross, gross anatomical examination. “All stains” denotes every staining procedure listed in Section 2.4.

**Table 3:** Gross topography and anatomical relationships of the orbital gland complex, presented separately for Kirk’s dik-dik and red forest duiker.

Species	Gland	Position in the Orbit	Gross Appearance	Main Anatomical Relationships	Excretory Duct/Drainage
Kirk’s dik-dik (*Madoqua kirkii*) n = 4 animals	**HG**	Medioventral region of the orbit	Oval gland with anterior and posterior extremities and dorsal and ventral borders	Located on the ventral rectus muscle; the posterior extremity was directed toward the orbital apex and was related to the ventral border of the SGTE and the ventral oblique muscle	A single excretory duct was observed on the medial surface of the gland
	**SGTE**	Medial part of the orbit	Oval, light pink, and grossly undivided gland	Located between the medial and ventral rectus muscles; partially covered by the ventral oblique muscle; closely associated with the third eyelid	The duct opening was not clearly identifiable macroscopically
	**LG**	Dorsolateral part of the orbit	Flat, triangular, light pink gland	Located between the dorsal and lateral rectus muscles	Small excretory ductules opened into the upper conjunctival fornix
Red forest duiker (*Cephalophorus natalensis*) n = 5 animals	**HG**	Medioventral region of the orbit	Oval gland with anterior and posterior extremities and dorsal and ventral borders	Located on the ventral rectus muscle; the posterior extremity was directed toward the orbital apex and was related to the ventral border of the SGTE and the ventral oblique muscle	A single excretory duct was observed on the medial surface of the gland
	**SGTE**	Medial part of the orbit	Oval, light pink, and grossly undivided gland	Located between the medial and ventral rectus muscles; partially covered by the ventral oblique muscle; closely associated with the third eyelid	The duct opening was not clearly identifiable macroscopically
	**LG**	Dorsolateral part of the orbit	Flat, triangular, light pink gland	Located between the dorsal and lateral rectus muscles	Small excretory ductules opened into the upper conjunctival fornix

Abbreviations: HG, Harderian gland; LG, lacrimal gland; SGTE, superficial gland of the third eyelid. No consistent differences in gross topography or general appearance were observed between the examined animals of the two species.

## Data Availability

The original contributions presented in this study are included in the article/Supplementary Material. Further inquiries can be directed to the corresponding authors.

## Supplementary Materials

The following supporting information can be downloaded at: https://www.mdpi.com/article/10.3390/vetsci13080752/s1, Figures S1–S9, showing the histochemical reactions of the HG, SGTE, and LG in Kirk’s dik-dik and red forest duiker. Figure S1. Representative histological features of the Harderian gland in young red forest duikers (Cephalophorus natalensis). (a) General organization of the gland, with a very thick connective tissue capsule and adipocytes associated with the capsule. (b) Thick interlobular septa dividing the glandular parenchyma into lobes; blood vessels and adipocytes are visible within the connective tissue. (c) Tubuloacinar organization, showing acini and tubules lined by secretory epithelium. (d) Modified Russell–Movat pentachrome staining showing mucosubstance-containing acini. (e) High-magnification view of myoepithelial cells and clusters of plasma cells in the glandular interstitium. Abbreviations: a, acini; ac, adipocytes; bv, blood vessels; c, capsule; is, interlobular septa; l, lobes; mec, myoepithelial cells; pc, plasma cells; tu, tubules. Staining: (a, c) Mayer’s hematoxylin and eosin; (b) Mallory trichrome with aniline blue; (d) modified Russell–Movat pentachrome; (e) methyl green–pyronin Y. Scale bars: (a) 100 µm; (b) 200 µm; (c, d) 50 µm; (e) 10 µm. Figure S2. Representative histological features of the superficial gland of the third eyelid in young red forest duikers (Cephalophorus natalensis). (a) General organization of the gland and its relationship with the cartilage of the third eyelid. (b) Thick connective tissue septa dividing the gland into small and medium-sized lobes. (c) Predominantly acinar secretory parenchyma with tubules and myoepithelial cells. (d) Modified Russell–Movat pentachrome staining showing numerous mucosubstance-containing secretory units. (e) High-magnification view of plasma cells and lymphocytes within the glandular interstitium. Abbreviations: a, acini; bv, blood vessels; c, capsule; cte, cartilage of the third eyelid; is, interlobular septa; l, lobes; lym, lymphocytes; mec, myoepithelial cells; pc, plasma cells; tu, tubules. Staining: (a, c) Mayer’s hematoxylin and eosin; (b) Mallory trichrome with aniline blue; (d) modified Russell–Movat pentachrome; (e) methyl green–pyronin Y. Scale bars: (a) 100 µm; (b) 200 µm; (c, d) 50 µm; (e) 10 µm. Figure S3. Representative histological features of the lacrimal gland in young red forest duikers (Cephalophorus natalensis). (a) General organization of the gland, with a thick connective tissue capsule. (b) Interlobular septa containing numerous blood vessels and dividing the parenchyma into large lobes. (c) Tubuloacinar organization with branched tubular components and demilune-like acinar terminal portions. (d) Modified Russell–Movat pentachrome staining showing abundant mucosubstance-containing secretory units. (e) High-magnification view of myoepithelial cells and scattered plasma cells within the glandular interstitium. Abbreviations: a, acini; bv, blood vessels; c, capsule; dl, demilune-like acinar terminal portions; is, in-terlobular septa; l, lobes; mec, myoepithelial cells; pc, plasma cells; tu, tubules. Staining: (a, c) Mayer’s hematoxylin and eosin; (b) Mallory trichrome with aniline blue; (d) modified Russell–Movat pentachrome; (e) methyl green–pyronin Y. Scale bars: (a) 100 µm; (b) 200 µm; (c, d) 50 µm; (e) 10 µm. Figure S4. Representative histochemical reactions in the secretory units and tubules of the Harderian gland in young Kirk’s dik-diks (Madoqua kirkii). Abbreviations: a, acini; tu, tubules. Staining: (a) PAS; (b) Alcian blue pH 1.0; (c) Alcian blue pH 2.5; (d) Alcian blue pH 2.5/PAS; (e) colloidal iron. Scale bars: (a–e) 20 µm. Figure S5. Representative histochemical reactions in the secretory units and tubules of the superficial gland of the third eyelid in young Kirk’s dik-diks (Madoqua kirkii). Abbreviations: a, acini; tu, tubules. Staining: (a) PAS; (b) Alcian blue pH 1.0; (c) Alcian blue pH 2.5; (d) Alcian blue pH 2.5/PAS; (e) colloidal iron. Scale bars: (a–e) 20 µm. Figure S6. Representative histochemical reactions in the secretory units and tubules of the lacrimal gland in young Kirk’s dik-diks (Madoqua kirkii). Abbreviations: a, acini; tu, tubules. Staining: (a) PAS; (b) Alcian blue pH 1.0; (c) Alcian blue pH 2.5; (d) Alcian blue pH 2.5/PAS; (e) colloidal iron. Scale bars: (a–e) 20 µm. Figure S7. Representative histochemical reactions in the secretory units and tubules of the Harderian gland in young red forest duikers (Cephalophorus natalensis). Abbreviations: a, acini; tu, tubules. Staining: (a) PAS; (b) Alcian blue pH 1.0; (c) Alcian blue pH 2.5; (d) Alcian blue pH 2.5/PAS; (e) colloidal iron. Scale bars: (a–e) 20 µm. Figure S8. Representative histochemical reactions in the secretory units and tubules of the superficial gland of the third eyelid in young red forest duikers (Cephalophorus natalensis). Abbreviations: a, acini; tu, tubules. Staining: (a) PAS; (b) Alcian blue pH 1.0; (c) Alcian blue pH 2.5; (d) Alcian blue pH 2.5/PAS; (e) colloidal iron. Scale bars: (a–e) 20 µm. Figure S9. Representative histochemical reactions in the secretory units and tubules of the lacrimal gland in young red forest duikers (Cephalophorus natalensis). Abbreviations: a, acini; tu, tubules. Staining: (a) PAS; (b) Alcian blue pH 1.0; (c) Alcian blue pH 2.5; (d) Alcian blue pH 2.5/PAS; (e) colloidal iron. Scale bars: (a–e) 20 µm.
