# Supplementary material for: Preliminary Anatomical, Histological, and Histochemical Study of the Orbital Gland Complex in Kirk’s Dik-Dik (Madoqua kirkii) and Red Forest Duiker (Cephalophorus natalensis)
Source: Vet Sci. 2026 Jul 29;13(8):752. doi: 10.3390/vetsci13080752 (PMC13517818; doi:10.3390/vetsci13080752)
Supplement: Supplementary file 1 [file vetsci-13-00752-s001.zip › vetsci-4405218-supplementary.pdf]

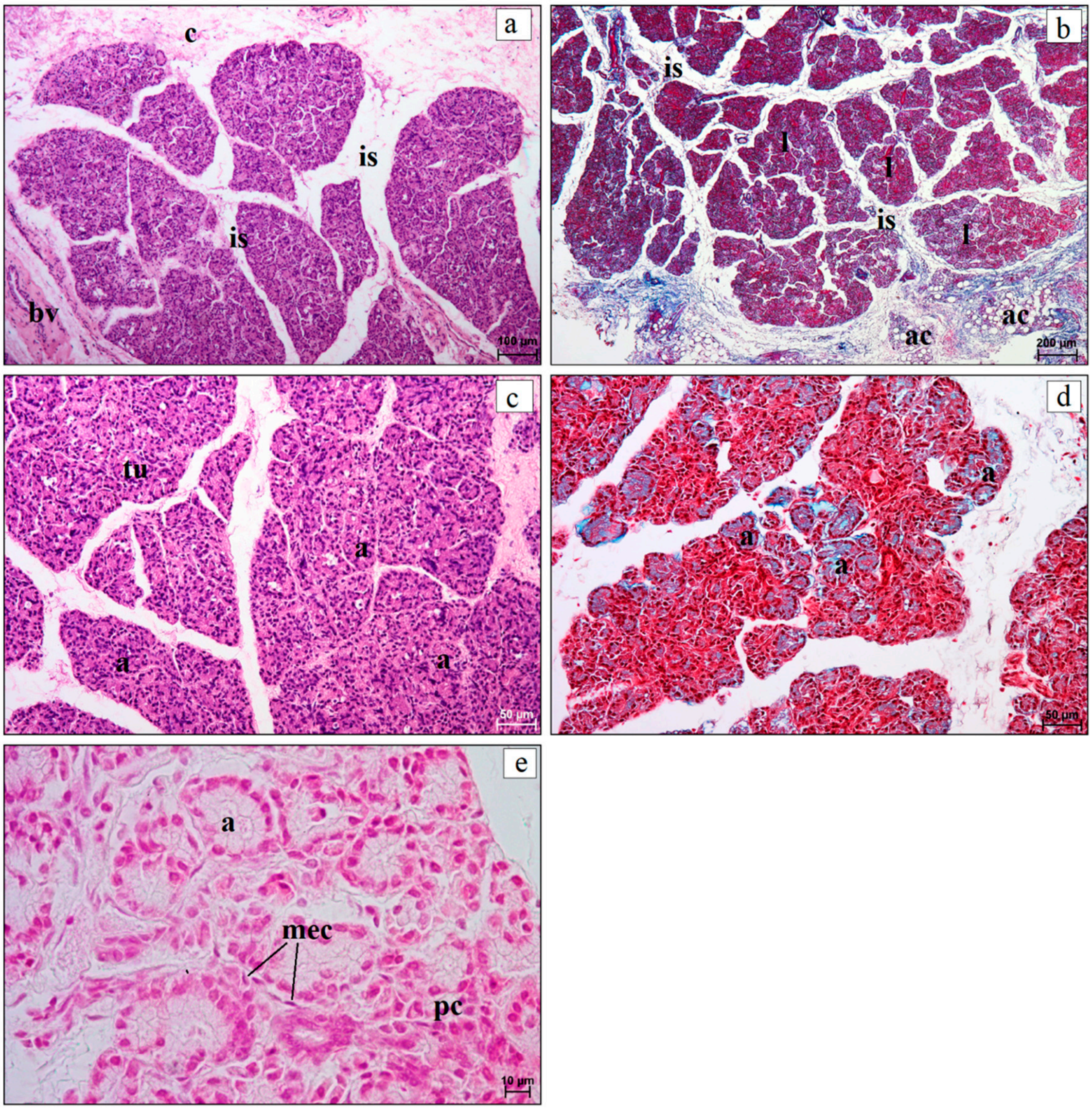


**Figure S1.**

Representative histological features of the Harderian gland in young red forest duikers (Cephalophorus natalensis). (a) General organization of the gland, with a very thick connective tissue capsule and adipocytes associated with the capsule. (b) Thick interlobular septa dividing the glandular parenchyma into lobes; blood vessels and adipocytes are visible within the connective tissue. (c) Tubuloacinar organization, showing acini and tubules lined by secretory epithelium. (d) Modified Russell–Movat pentachrome staining showing mucosubstance-containing acini. (e) High-magnification view of myoepithelial cells and clusters of plasma cells in the glandular interstitium. Abbreviations: a, acini; ac, adipocytes;

bv, blood vessels; c, capsule; is, interlobular septa; l, lobes; mec, myoepithelial cells; pc, plasma cells; tu, tubules. Staining: (a, c) Mayer's hematoxylin and eosin; (b) Mallory trichrome with aniline blue; (d) modified Russell–Movat pentachrome; (e) methyl green–pyronin Y. Scale bars: (a) 100 µm; (b) 200 µm; (c, d) 50 µm; (e) 10 µm.

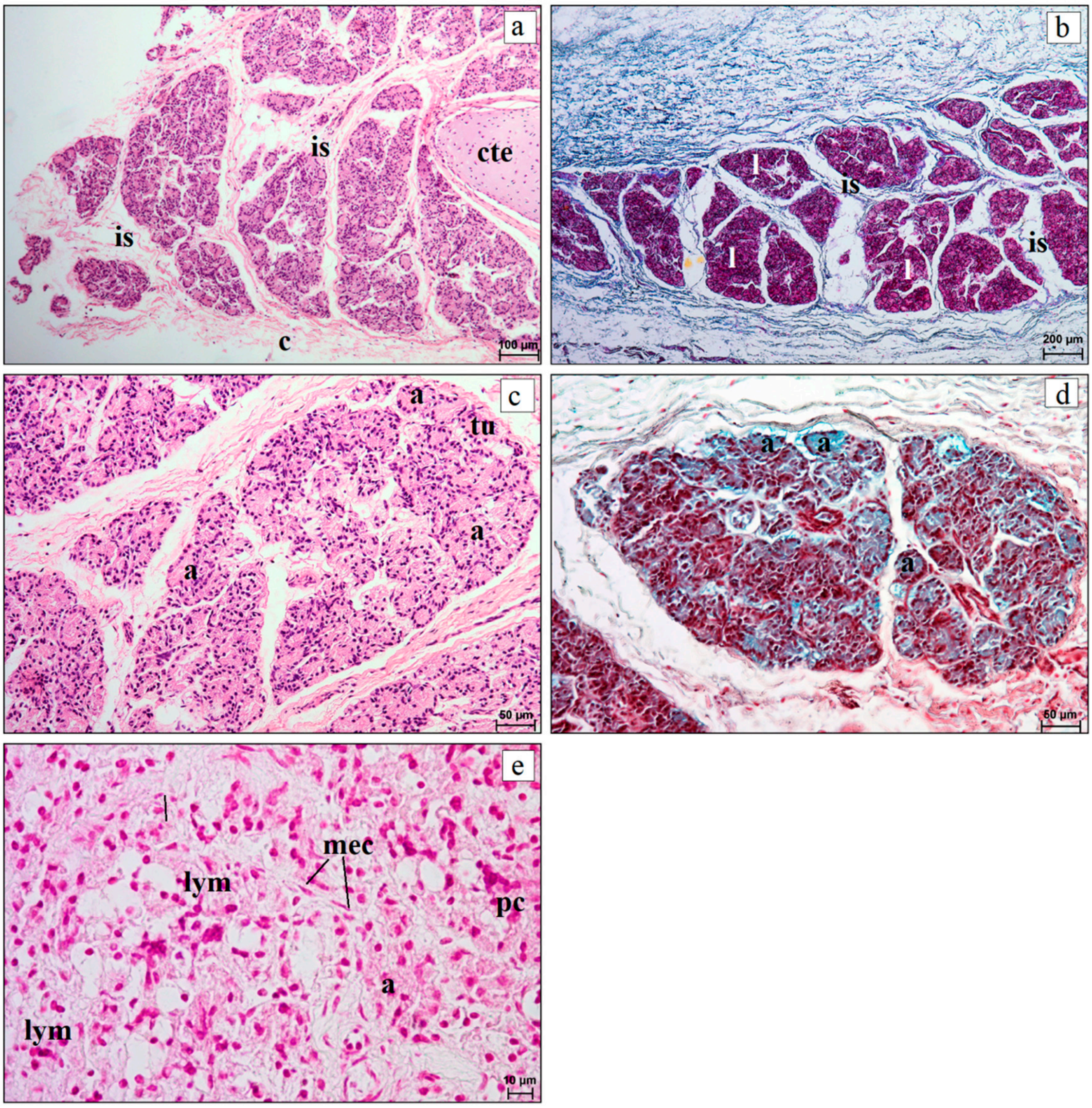


**Figure S2.**

Representative histological features of the superficial gland of the third eyelid in young red forest duikers (Cephalophorus natalensis). (a) General organization of the gland and its relationship with the cartilage of the third eyelid. (b) Thick connective tissue septa dividing

the gland into small and medium-sized lobes. (c) Predominantly acinar secretory parenchyma with tubules and myoepithelial cells. (d) Modified Russell–Movat pentachrome staining showing numerous mucosubstance-containing secretory units. (e) High-magnification view of plasma cells and lymphocytes within the glandular interstitium. Abbreviations: a, acini; bv, blood vessels; c, capsule; cte, cartilage of the third eyelid; is, interlobular septa; l, lobes; lym, lymphocytes; mec, myoepithelial cells; pc, plasma cells; tu, tubules. Staining: (a, c) Mayer's hematoxylin and eosin; (b) Mallory trichrome with aniline blue; (d) modified Russell–Movat pentachrome; (e) methyl green–pyronin Y. Scale bars: (a) 100 μm; (b) 200 μm; (c, d) 50 μm; (e) 10 μm.

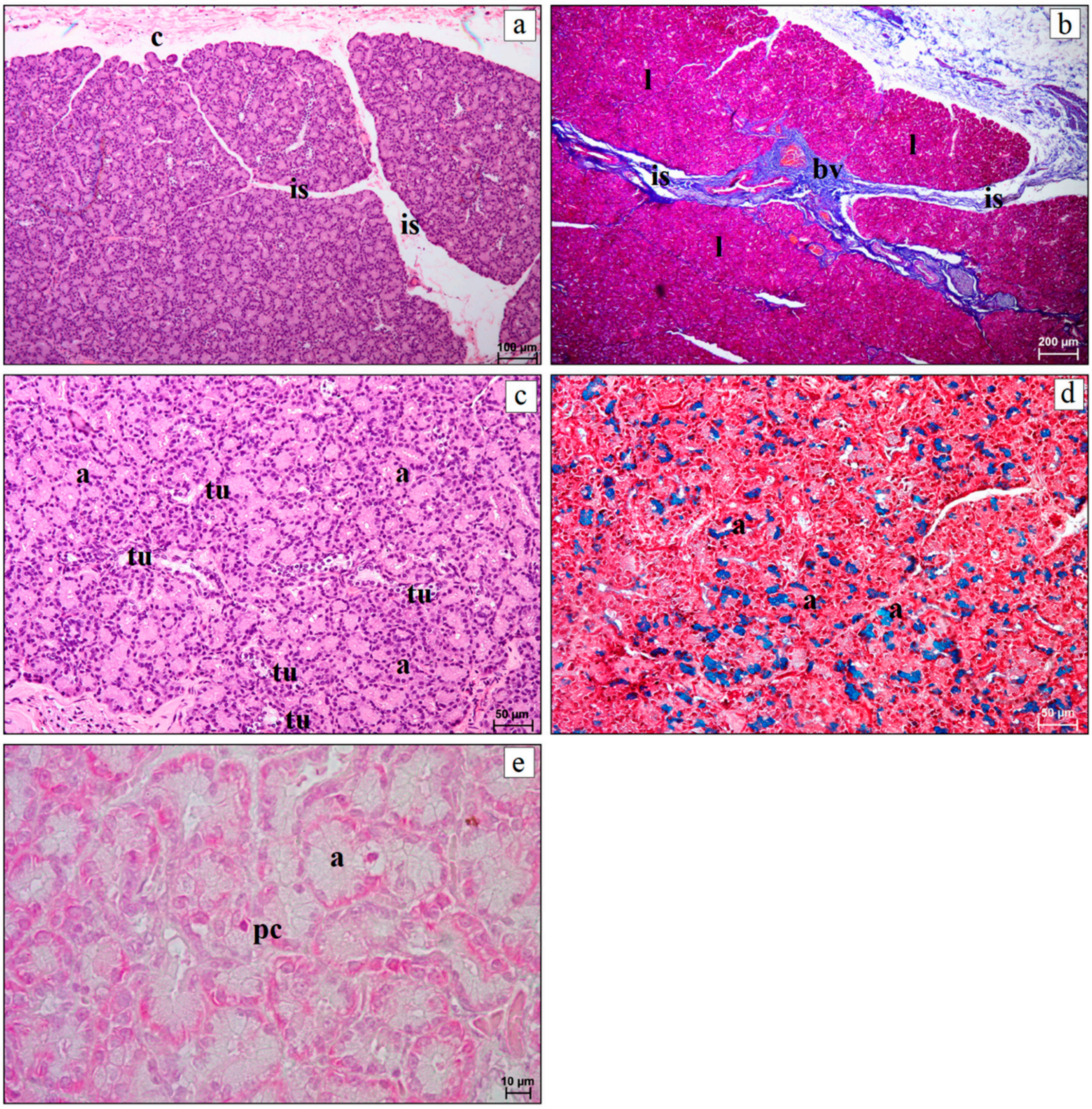

**Figure S3.**

Representative histological features of the lacrimal gland in young red forest duikers (Cephalophorus natalensis). (a) General organization of the gland, with a thick connective tissue capsule. (b) Interlobular septa containing numerous blood vessels and dividing the parenchyma into large lobes. (c) Tubuloacinar organization with branched tubular components and demilune-like acinar terminal portions. (d) Modified Russell–Movat pentachrome staining showing abundant mucosubstance-containing secretory units. (e) High-magnification view of myoepithelial cells and scattered plasma cells within the glandular interstitium. Abbreviations: a, acini; bv, blood vessels; c, capsule; dl, demilune-like acinar terminal portions; is, in-terlobular septa; l, lobes; mec, myoepithelial cells; pc, plasma cells; tu, tubules. Staining: (a, c) Mayer's hematoxylin and eosin; (b) Mallory trichrome with aniline blue; (d) modified Russell–Movat pentachrome; (e) methyl green–pyronin Y. Scale bars: (a) 100 µm; (b) 200 µm; (c, d) 50 µm; (e) 10 µm.

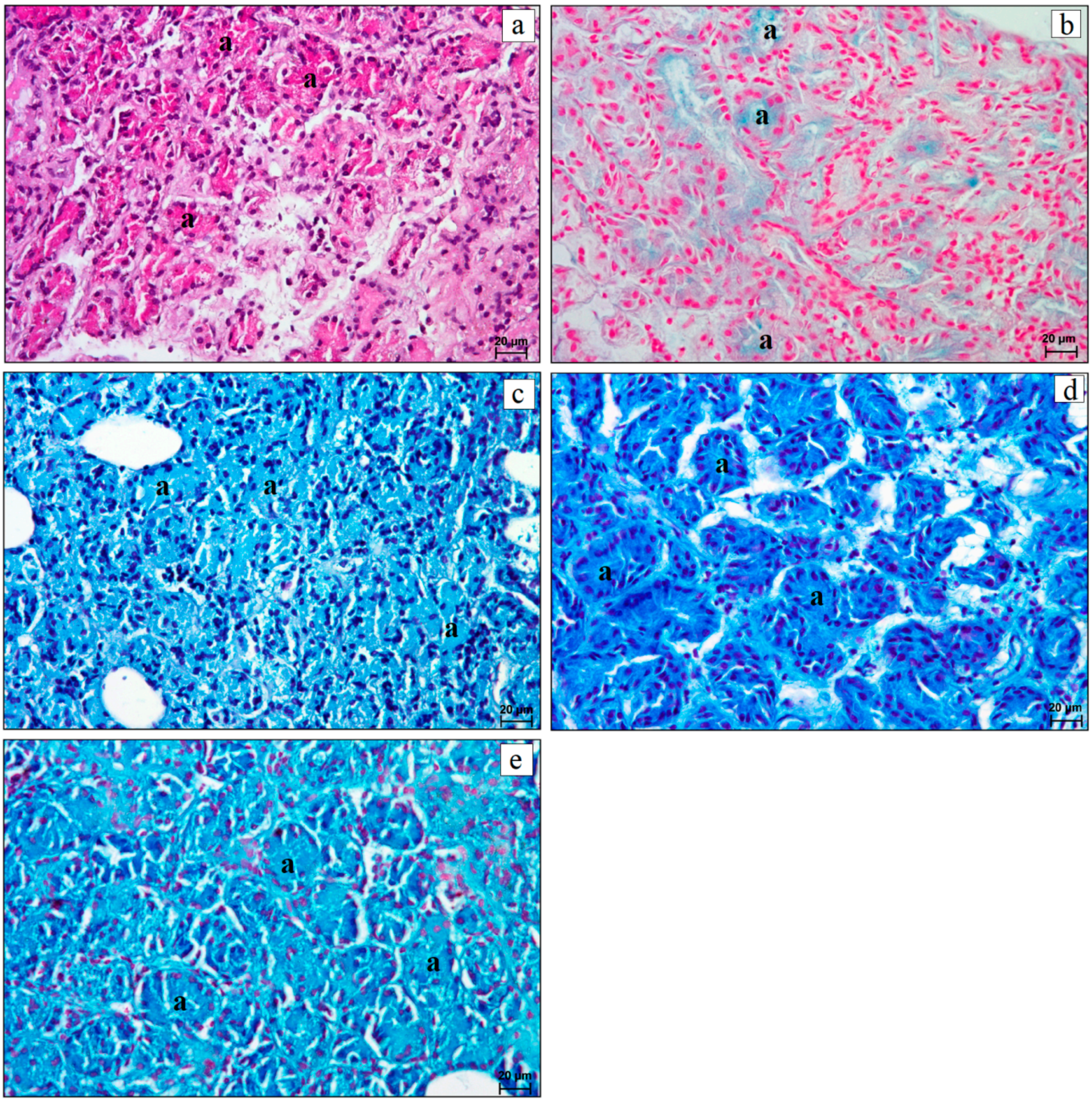


**Figure S4.**

Representative histochemical reactions in the secretory units and tubules of the Harderian gland in young Kirk's dik-diks (*Madoqua kirkii*).

Abbreviations: a, acini; tu, tubules.

Staining: (a) PAS; (b) Alcian blue pH 1.0; (c) Alcian blue pH 2.5; (d) Alcian blue pH 2.5/PAS; (e) colloidal iron.

Scale bars: (a–e) 20 µm.

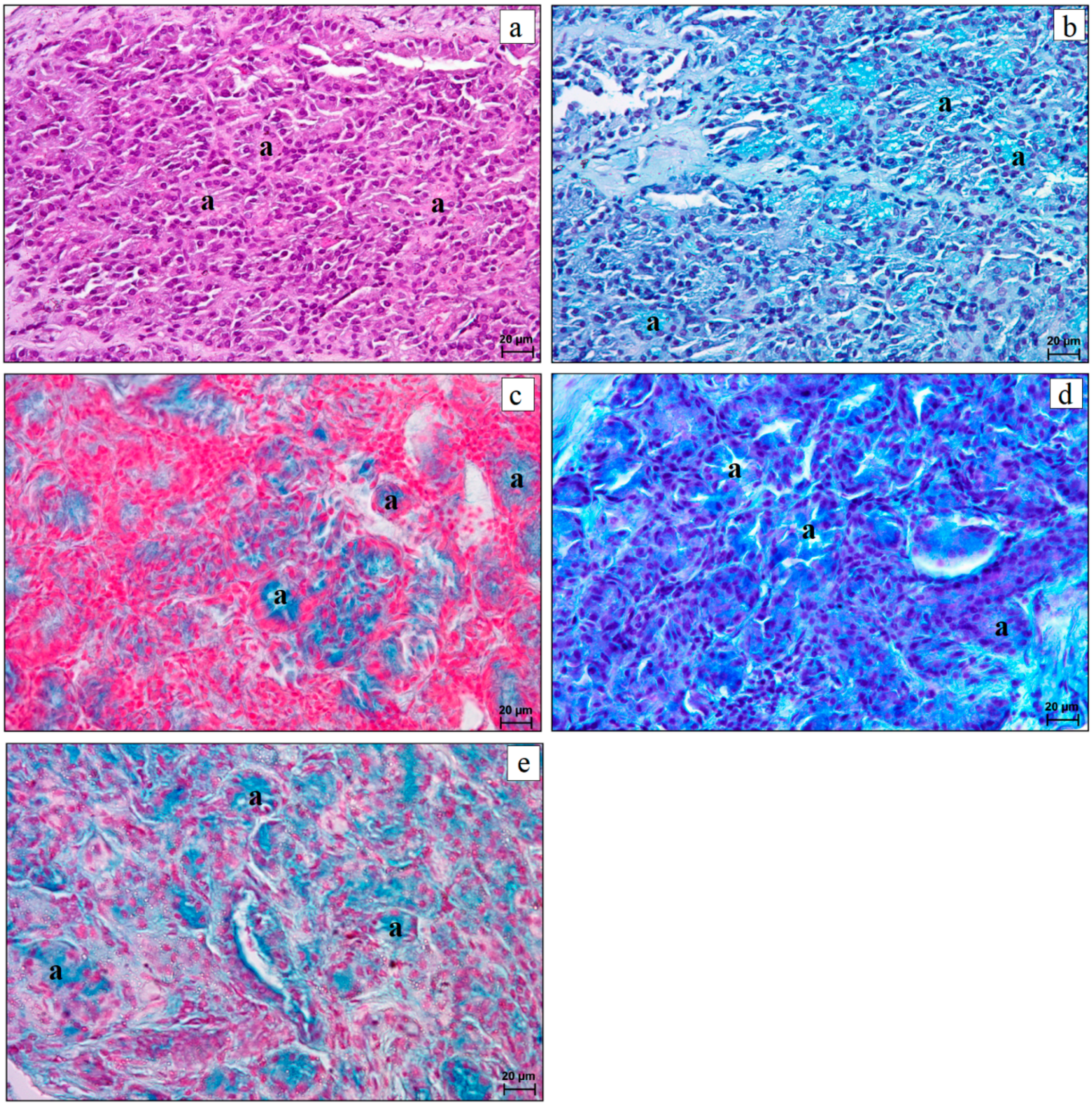


**Figure S5.**

Representative histochemical reactions in the secretory units and tubules of the superficial gland of the third eyelid in young Kirk's dik-diks (*Madoqua kirkii*).

Abbreviations: a, acini; tu, tubules.

Staining: (a) PAS; (b) Alcian blue pH 1.0; (c) Alcian blue pH 2.5; (d) Alcian blue pH 2.5/PAS; (e) colloidal iron.

Scale bars: (a–e) 20 µm.

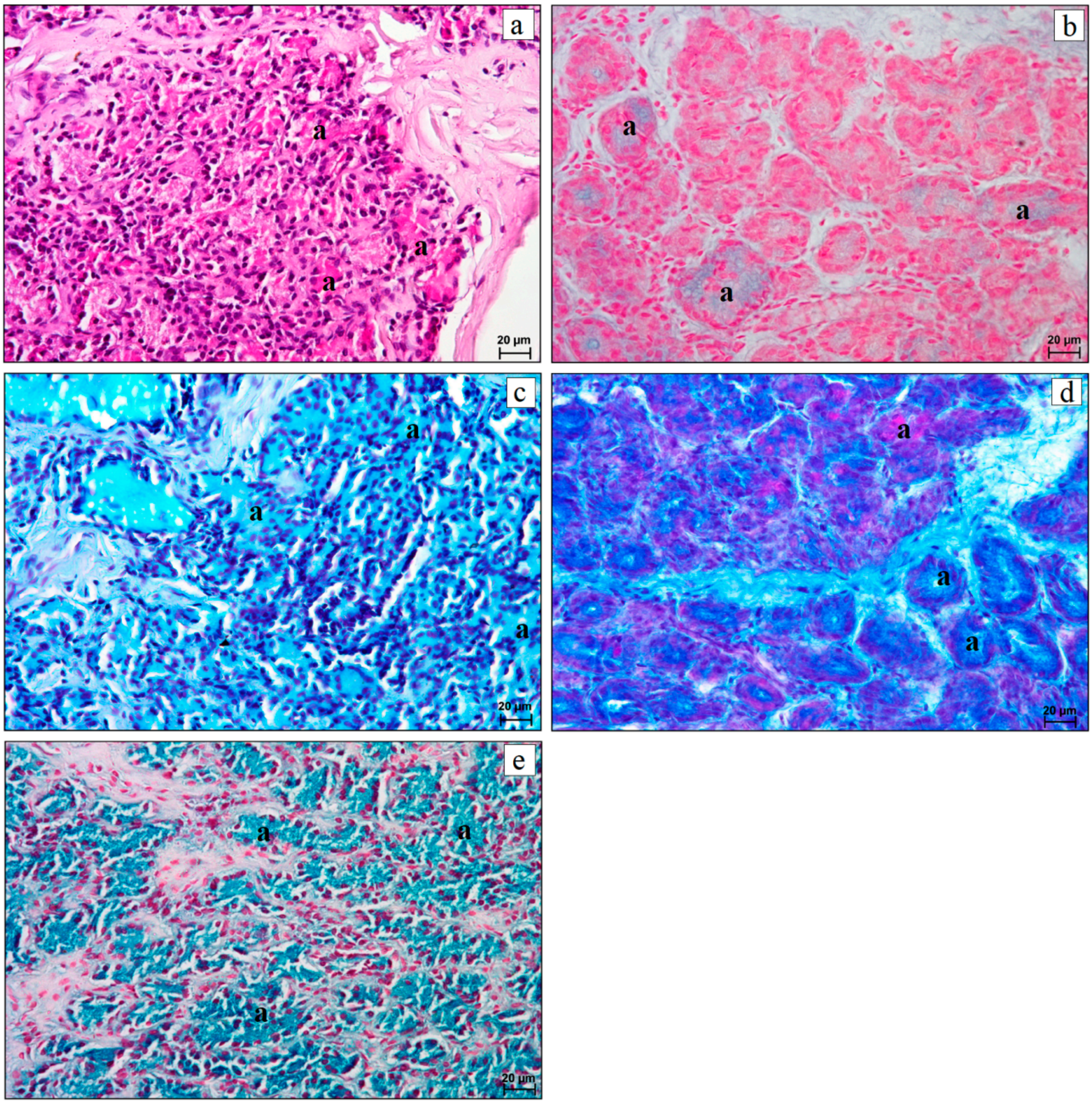


**Figure S6.**

Representative histochemical reactions in the secretory units and tubules of the lacrimal gland in young Kirk's dik-diks (*Madoqua kirkii*).

Abbreviations: a, acini; tu, tubules.

Staining: (a) PAS; (b) Alcian blue pH 1.0; (c) Alcian blue pH 2.5; (d) Alcian blue pH 2.5/PAS; (e) colloidal iron.

Scale bars: (a–e) 20 µm.

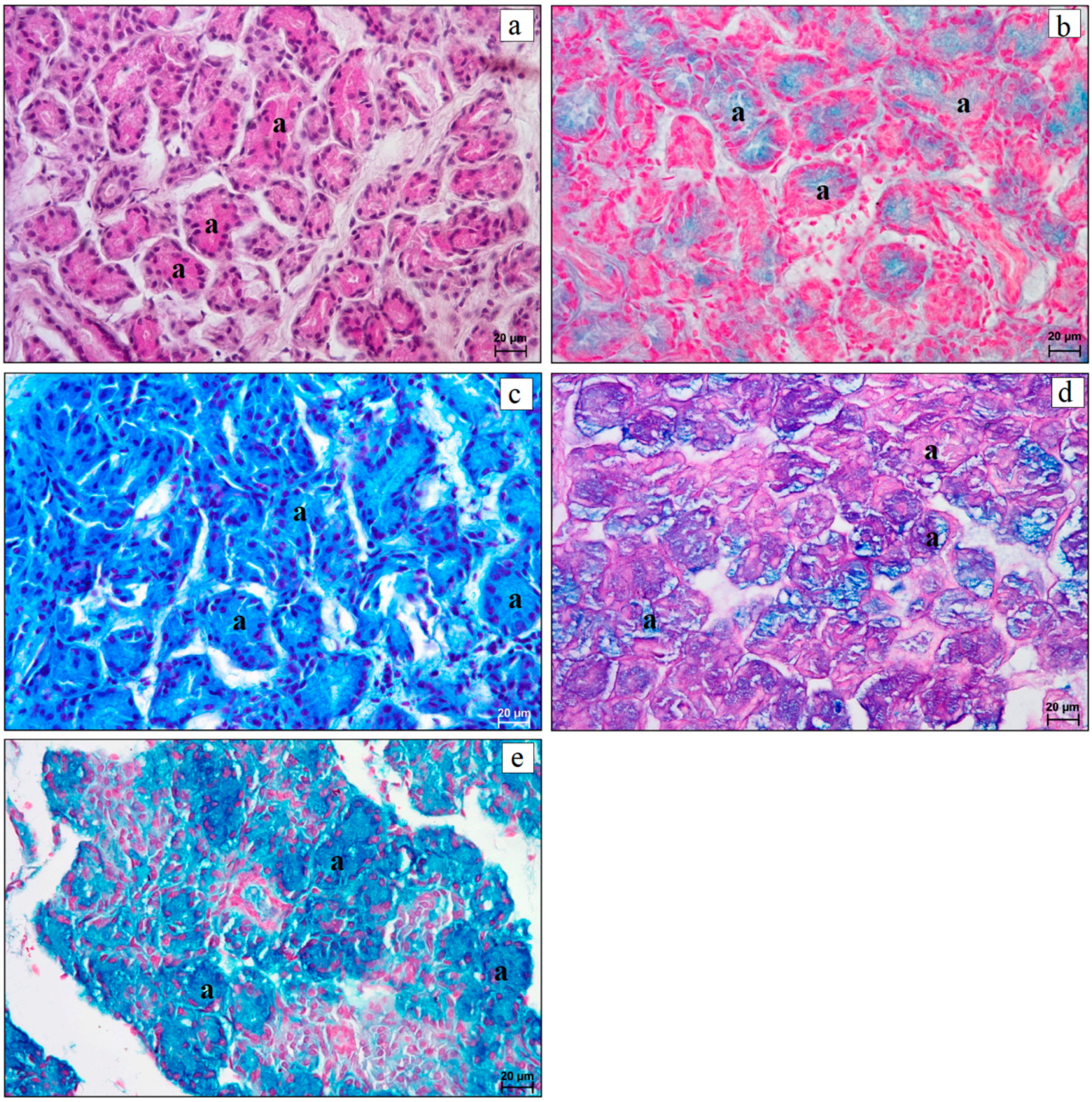


**Figure S7.**

Representative histochemical reactions in the secretory units and tubules of the Harderian gland in young red forest duikers (*Cephalophorus natalensis*).

Abbreviations: a, acini; tu, tubules.

Staining: (a) PAS; (b) Alcian blue pH 1.0; (c) Alcian blue pH 2.5; (d) Alcian blue pH 2.5/PAS; (e) colloidal iron.

Scale bars: (a–e) 20 µm.

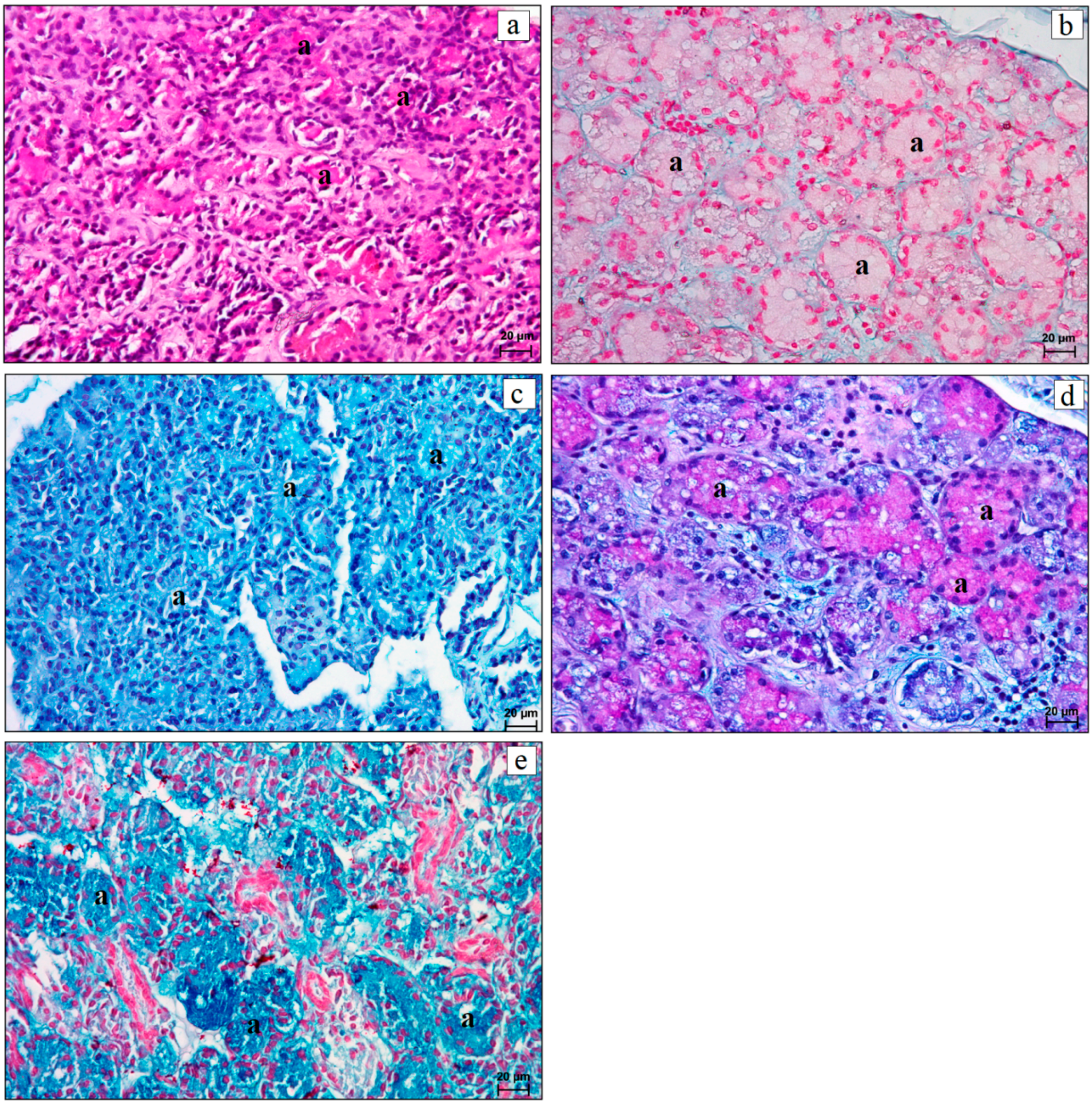


**Figure S8.**

Representative histochemical reactions in the secretory units and tubules of the superficial gland of the third eyelid in young red forest duikers (*Cephalophorus natalensis*).

Abbreviations: a, acini; tu, tubules.

Staining: (a) PAS; (b) Alcian blue pH 1.0; (c) Alcian blue pH 2.5; (d) Alcian blue pH 2.5/PAS; (e) colloidal iron.

Scale bars: (a–e) 20 µm.

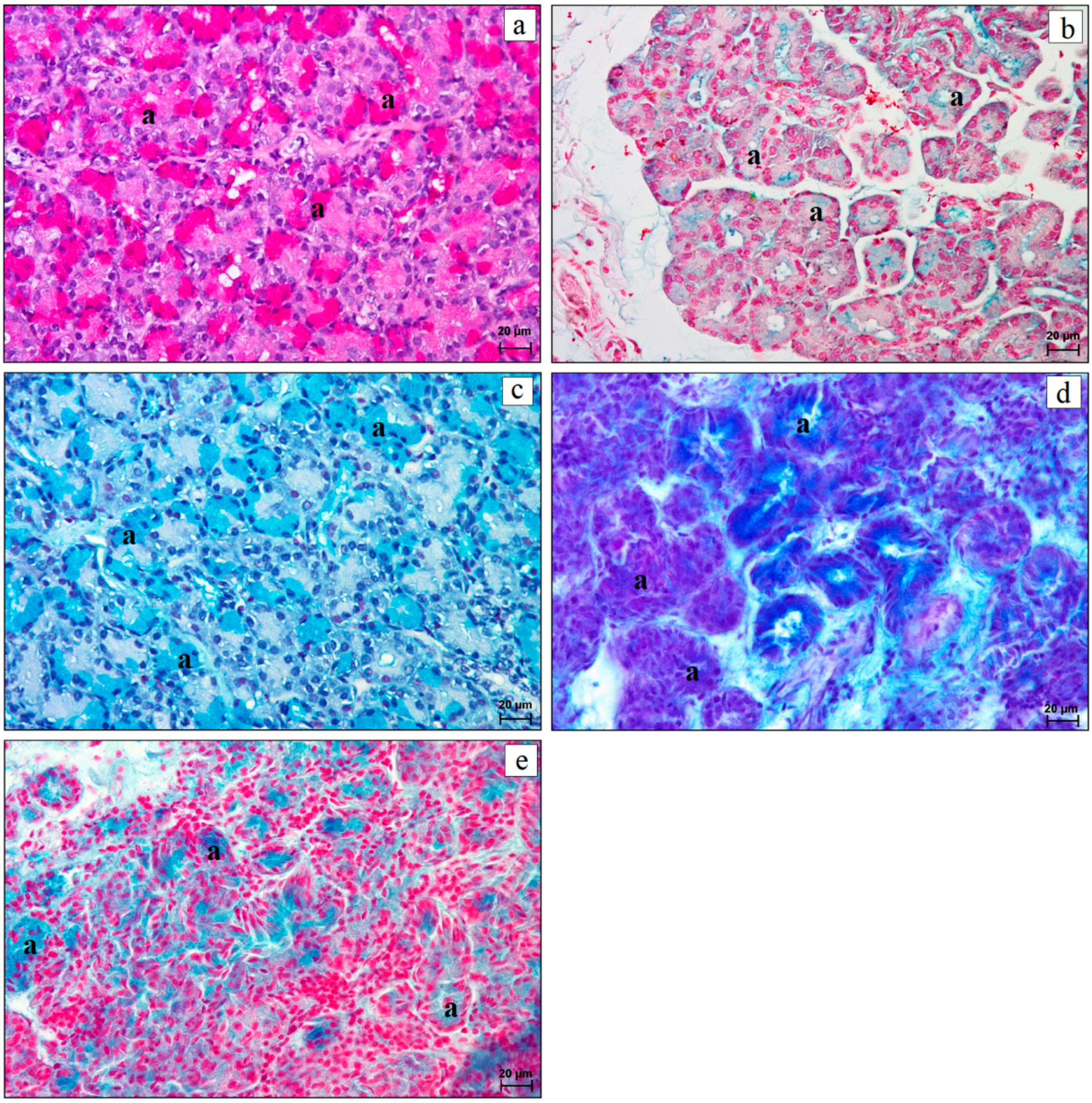


**Figure S9.**

Representative histochemical reactions in the secretory units and tubules of the lacrimal gland in young red forest duikers (*Cephalophorus natalensis*).

Abbreviations: a, acini; tu, tubules.

Staining: (a) PAS; (b) Alcian blue pH 1.0; (c) Alcian blue pH 2.5; (d) Alcian blue pH 2.5/PAS; (e) colloidal iron.

Scale bars: (a–e) 20 µm.
